# Everything’s under Control: Maximizing Biosensor Performance through Negative Control Probe Selection

**DOI:** 10.1021/acs.analchem.4c05854

**Published:** 2025-02-03

**Authors:** Joseph Bucukovski, Benjamin L. Miller

**Affiliations:** a Department of Biochemistry and Biophysics, University of Rochester, Rochester, New York 14627, United States; b Institute of Optics, University of Rochester, Rochester, New York 14627, United States; c Department of Dermatology, University of Rochester, Rochester, New York 14627, United States; d Program in Materials Science, University of Rochester, Rochester, New York 14627, United States; e Department of Biomedical Engineering, University of Rochester, Rochester, New York 14627, United States

## Abstract

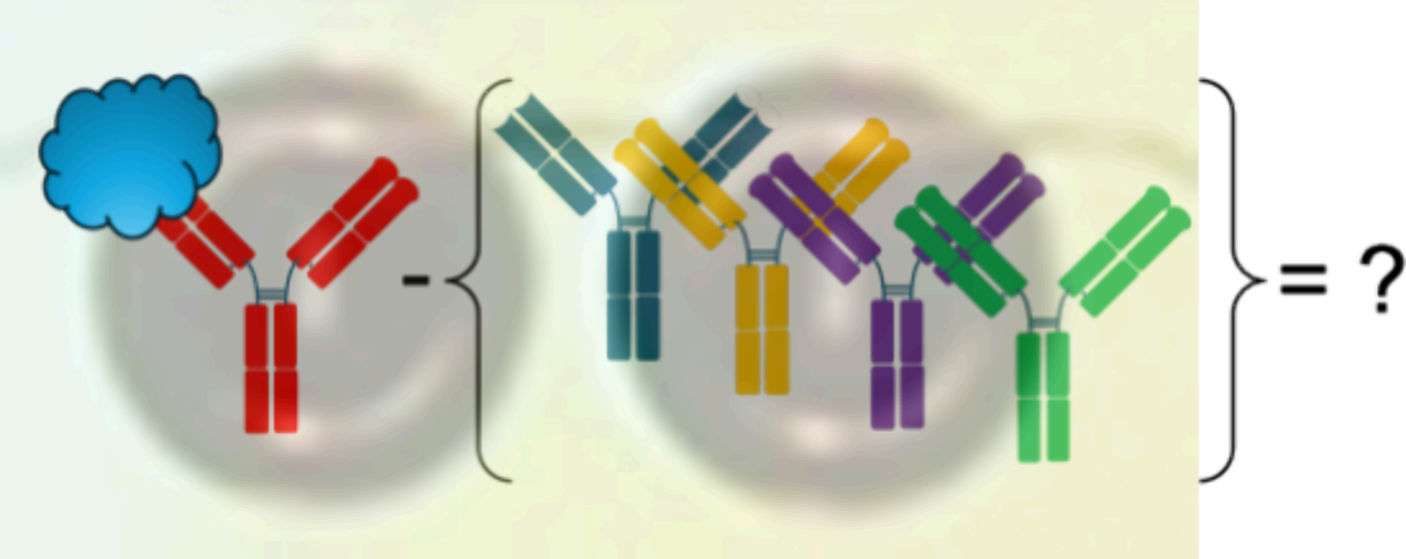

The rapid rise of label-free biosensing technologies has led to multiple creative strategies for the detection of macromolecules in complex biological solutions for disease state monitoring, drug discovery, and basic science research. A challenge with these techniques is that assays conducted in complex media such as serum suffer from nonspecific binding of matrix constituents. In label-free biosensors, it is virtually impossible to distinguish these nonspecific interactions without the use of a reference (negative control) probe. Only with reference subtraction can the specific binding signal be faithfully reported. To date, this has been a sparsely studied area in the biosensing field. Here, we report an FDA-inspired framework for optimum control probe selection and a systematic analysis for determining the optimal negative control probe given two monoclonal antibody capture probes on photonic ring resonator sensors. Briefly, while the differences in assay performance for IL-17A and CRP were found to be subtle, the best-scoring reference control based on the bioanalytical parameters of linearity, accuracy, and selectivity differed for each analyte. In the IL-17A assay, BSA scored the highest at 83%, while mouse IgG1 isotype control antibody placed a close second with 75%. With respect to the CRP assay, the rat IgG1 isotype control antibody scored the highest at 95%, while anti-FITC scored the second highest at 89%. These results suggest that although isotype-matching to the capture antibody may be tempting, the best on-chip reference control must be optimized on a case-by-case basis using the framework we report.

## Introduction

**Figure 1:**
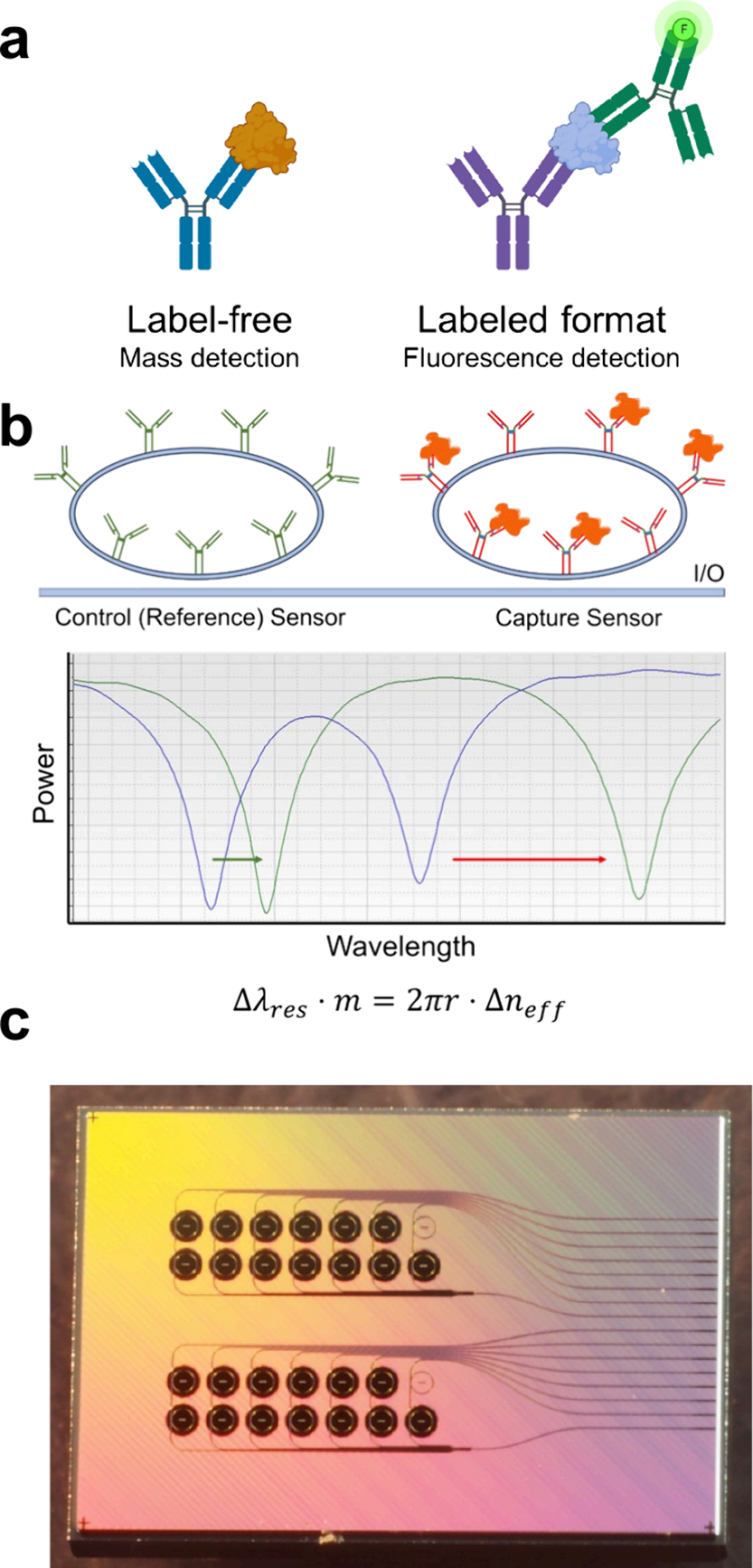
(a) Schematic of labeled vs label-free assays. (b) While applicable to any sensor type, this study used ring resonators. Each bus waveguide interfaced with two rings: one control (reference), and one capture (experimental). By subtracting resonance shifts (shown in the graph) produced by a control ring from those produced by a ring functionalized with an antibody to the target, the specific binding signal is obtained. The equation below the image describes the operation of the sensor, in which changes in the local refractive index (Δ*n*_eff_) cause changes in the resonant wavelength (Δλ_res_). (c) Photograph of one of the ring resonator photonic integrated circuit (PIC) chips used in this work.

Biosensors are analytical devices that provide information about a biological sample, such as the concentration of a specific analyte or set of analytes. Immunosensors, nucleic acid-, enzyme-, and whole cell-based sensors must include a biorecognition element and a signal transducer unless they are strictly spectroscopy based.^1−3^ Biorecognition elements (bioreceptors) may include antibodies, aptamers, and DNA oligonucleotides that can selectively bind the target analyte of interest. Signal transducers can be optical, electrochemical, or mechanical methods that take advantage of changes in the physicochemical properties of the sensing element as a function of target molecule binding. Methods measuring changes in mass or refractive index are commonly referred to as *label-free*, while those requiring additional chemical reagents to detect a species are *labeled* transducers (Figure 1A). In labeled “turn-on” biosensors, transduction occurs following a bioreceptor–ligand binding event and a signal, such as fluorescence or chemiluminescence, is generated by a labeled 2° reagent.^4^ In “label-free” biosensors, binding of the target analyte to the bioreceptor directly leads to production of a signal, without additional reagents. In both cases, the sensor response that is generated is ideally specific for the target of interest, and directly proportional to the amount of target bound to the bioreceptor. Where this relationship is nonlinear, sensor calibration can determine the mathematical model relating analyte concentration and response.

Unfortunately, label-free sensors are subject to error caused by nonspecific binding (NSB): binding of nontarget analytes to the bioreceptor sensing area. Electrostatics, hydrogen bonding, and van der Waals interactions all contribute to NSB.^5,6^ Of particular importance when considering electrostatic interactions is the pH of the solution. The closer to the protein’s isoelectric point (pI) the pH lies, the more neutrally charged the protein, potentially increasing NSB due to hydrophobic interactions. The matrix also influences the extent to which NSB is observed. For example, the measurement of an analyte in human serum will result in more NSB than measuring that same analyte in buffer, for the simple reason that there are more species to bind nonspecifically. Serum proteins are notorious for binding nonspecifically to bioreceptors and the underlying sensor substrate, increasing the level of noise or error in the assay.^7^ Labeled assays have the benefit of amplifying the analyte response, and in principle are less impacted by NSB. However, such reagents are expensive, increase assay complexity, and are incompatible with real-time sensing.

One strategy for dealing with NSB is to implement a reference channel to subtract the nonspecific binding contribution in the overall assay response. For example, surface plasmon resonance (SPR) experiments typically involve immobilizing a noninteracting or mutant biomolecule of the same class and density as the ligand (capture probe) in the reference flow cell to remove NSB contributions from the overall binding curve.^8,9^ Alternatively, other SPR users leave the reference cell blank with only the underlying functional chemistry present. This approach assumes that the effects of nonspecific binding to the ligand are negligible compared to the bulk refractive index (RI) shifts that are observed.^10,11^ Among many other examples, graphene field-effect transistor (gFET) biosensors have been used to capture exosomes via anti-CD63 monoclonal antibodies paired with an isotype-matched mouse IgG1κ antibody to correct for NSB, while AC impedimetric biosensors were shown to detect digoxin in buffer following reference subtraction from the nonspecific IgG antibody control signal.^12,13^ Combinations of specificity and background controls have been implemented,^14^ and isotype matching is commonly employed in mass cytometry and immunocytochemistry.^5,15^

Despite the above examples, properly vetted reference (negative control) probes are seldom reported in the literature, and there has been no systematic analysis of the effect of control probe selection on analytical performance as far as we are aware. While there can be no “perfect” control for NSB, such an analysis would be highly useful in designing new assays. Here, we report studies evaluating the impact of control probe selection on assay performance for photonic microring resonators (PhRR), a type of label-free optical biosensor.

PhRRs measure changes in refractive index of the surrounding medium (Figure 1b). Capture of an analyte increases the local effective refractive index due to displacement of the aqueous medium and produces a red-shift in the resonant wavelength.^16−18^ This principle is used to quantify the effect of increasing concentrations of target protein over the sensor surface allowing the generation of response (calibration) curves. For this study, PhRR sensor chips (photonic integrated circuits, or PICs) were fabricated at wafer scale in silicon nitride using 300 mm CMOS processes and demonstrate a bulk sensitivity up to 220 nm/RIU, which is consistent with the state of the art for PhRRs employed in biological sensing.^19−21^ The layout of each PIC was such that 13 individual sensors were accessible for functionalization and multiplex detection (Figure 1c).

PhRR sensors were functionalized with a panel of negative control proteins paired with anti-interleukin 17A (anti-IL-17A) or anti-C-Reactive Protein (anti-CRP) capture antibodies on a single PIC. Both IL-17A and CRP are important biomarkers of human disease and serve as representative case studies for other biomarkers of interest. IL-17A is an important pro-inflammatory cytokine of intermediate size (35 kDa) that exists as a homodimer.^22^ Its reference range in human serum is 0.5 to 15 pg/mL.^23^ CRP is a large (120 kDa) pentameric protein that is elevated to concentrations as much as 200 μg/mL in disease states such as sepsis.^24−26^ For control proteins, we hypothesized that pairing the capture antibody with a matched isotype control would enable subtraction of nonspecific binding without over- or under-correction of the real binding response.^12^ In addition to the isotype matched control antibody,^23,13^ we included 6 other candidate proteins in our negative control (reference) panel: 2 additional mouse (nonmatched) isotype control IgG, bovine serum albumin (BSA), antifluorescein isothiocyanate (anti-FITC), rat isotype control IgG1, and cytochrome c. The basis of the panel selection, aside from isotype matching, was to include biomolecules commonly found in serum, standard blocking reagents, nonspecific IgG, and charged nonantibody proteins.

In past work, we have used anti-FITC^27,28^ as a control probe as FITC dye is not normally found in biofluids. We^29,30^ and others have also found BSA to be a useful control. Isotype-matched antibody reference controls^31^ and mouse IgG have been used as well.^32,33^ Finally, mouse monoclonal capture probes have been paired with a polyclonal mouse IgG reference probe. This controls for isotype, but not for class and clonality.^34−36^

## Methods

### Material Sources

Anti-CRP (mouse IgG2b, monoclonal), rat IgG1, mouse IgG1, IgG2a, and IgG2b isotype control antibodies and recombinant human CRP were all purchased from R&D Systems. The antibodies were supplied as lyophilized material and reconstituted to 1.0 mg/mL in sterile PBS. CRP antigen was supplied as lyophilized material and reconstituted to the recommended concentration using sterile 20 mM Tris-HCl. Anti-fluorescein isothiocyanate (FITC; goat polyclonal) and bovine serum albumin (BSA) were purchased from Rockland Immunochemicals, while equine heart cytochrome c was purchased from MP Biomedicals, LLC. BSA and cytochrome c were supplied as lyophilized material and reconstituted to recommended concentrations in sterile PBS. Anti-FITC was supplied as a liquid on ice. Anti-IL-17A (mouse IgG1, monoclonal) and recombinant human IL-17A were purchased from BioLegend, Inc., and were supplied as a liquid on ice. PBS-T was prepared as phosphate buffered saline (10 mM monobasic sodium phosphate, 10 mM dibasic sodium phosphate, 150 mM NaCl) with 0.01% w/v Tween-20. Extracellular growth medium-2 (EGM-2) was obtained from Lonza and used as an assay diluent, along with fetal bovine serum (FBS) from Gibco diluted to 1% v/v.

*Photonic sensors, sensor functionalization, and probe/control protein deposition* were conducted as in our prior work. Detailed information is provided in the Supporting Information.

### Assembly of Microfluidic Devices

**Figure 2:**
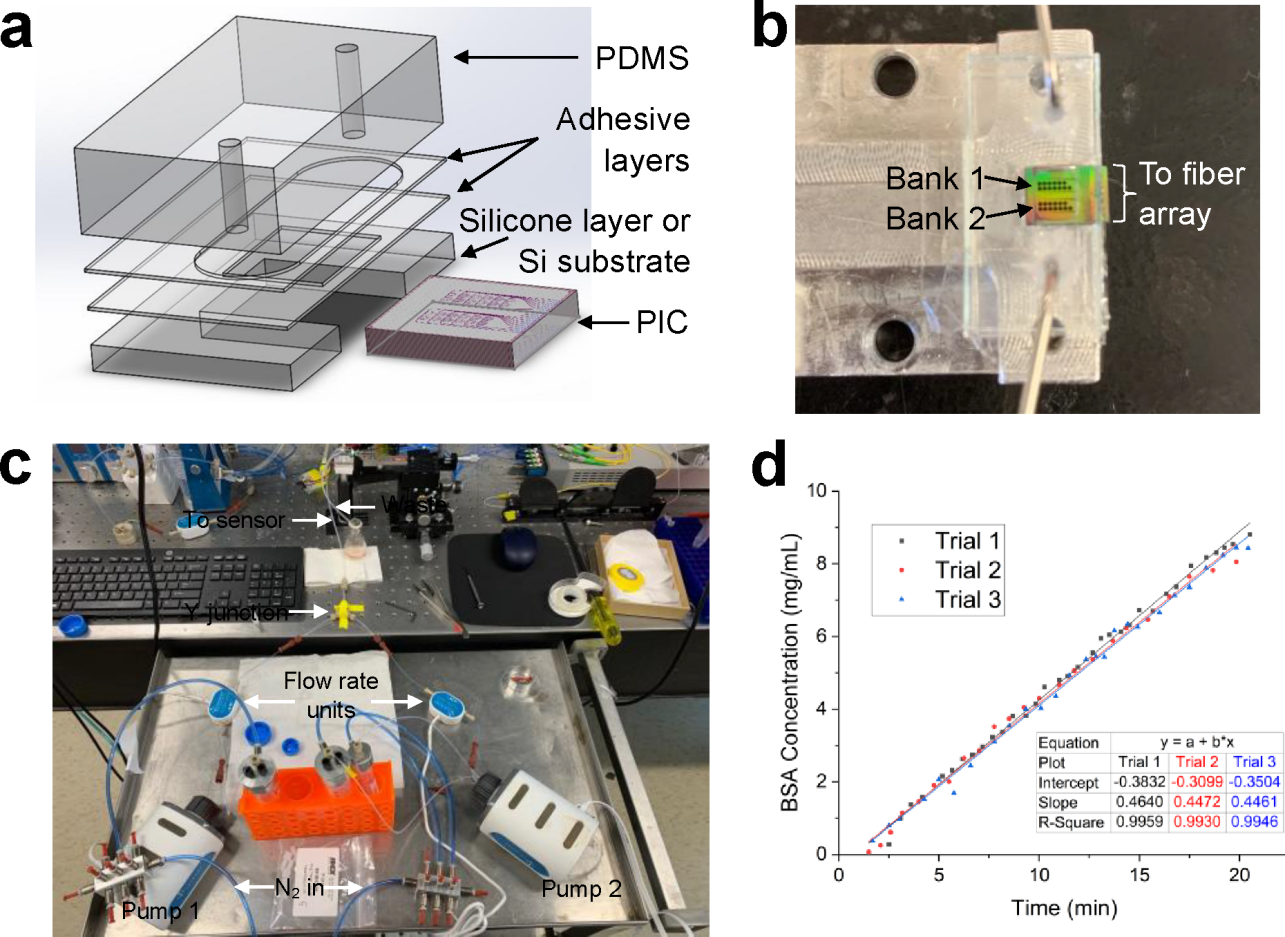
(a) Schematic of layered microfluidic devices used for all sensing experiments. (b) Top-down view of biosensor PIC packaged into a microfluidic device with both ring banks shown. All waveguides were accessed using edge-coupled 16 channel fiber arrays. (c) Overall construction of the microfluidic gradient generator. (d) The resulting concentrations of BSA output from the gradient generator were verified by A_280_ (Nanodrop) and plotted against time; *N* = 3.

PhRR sensor PICs were packaged in microfluidic devices as shown schematically in Figure 2a. This was accomplished with the use of pressure sensitive adhesive (PSA) from 3M and poly(dimethylsiloxane) (PDMS) elastomer synthesized with a base to curing ratio of 7.5 to 1. Briefly, 57 and 127 μm thick PSA was cut into specific patterns using a craft cutter (Silhouette Cameo). After inserting the PhRR PIC into the silicon chip holder layer and mounting onto a glass slide, the PSA sealing layer (57 μm) was adhered onto the assembly, exposing only the ring resonator sensors. Next, the PSA microfluidic channel layer (127 μm) was adhered onto the sealing layer to create a stack that opens fluidic access to the sensors. After UV-ozone treatment for 10 min, the PDMS gasket with precut inlet and outlet fluidic ports was affixed and bonded to the PSA channel layer to close the system. Prior to running analyte detection assays under flow, this assembly was mounted onto an aluminum temperature-controlled stage (Figure 2b) and was configured with 0.02” ID flexible tubing (Cole-Parmer, Masterflex) that was fitted with a 21-gauge metal elbow connector (Jansen) for insertion directly into the PDMS.

### PhRR Gradient Assays

The gradient generator (Figure 2) and optical apparatus used for all measurements are described in Supporting Information. For IL-17A detection assays, IL-17A was formulated to a concentration of 1 μg/mL in either 1% BSA in PBS-T, 1% BSA in EGM-2, or 1% FBS in PBS-T. The same diluent backgrounds were used for CRP detection assays, apart from the added condition of 20% FBS in assay wash buffer (PBS-ET), where the CRP analyte was formulated to a concentration of 5 μg/mL. The difference in FBS concentration was due to initial experiments suggesting assay interference in the IL-17A experiments when 20% FBS was used. For all gradient experiments, the analyte reservoir was designated as pump 2 of the gradient generator, while the buffer reservoir (diluent-matched) was assigned as pump 1. During equilibration, a 16-channel optical fiber array was used to couple light into the edge waveguides of the PhRR PIC overhanging from the microfluidic device. Optical alignment was optimized to maximize output power through each channel on the detector (Keysight, N7745C). Both pumps were primed with the appropriate target assay solutions (diluent and analyte) to mitigate air bubbles in the lines. The mixing T was connected to the gradient generator setup and flushed with diluent buffer. Then, diluent was flowed at 30 μL/min into the PIC microfluidic device to block the sensor surface for 30 min. Next, the program to initiate the linearly increasing concentration of IL-17A or CRP was started, and run for 20 min, with 2 additional minutes required to account for the dead volume of the tubing. Flow rates on each pump were carefully monitored and recorded at the end of the program, along with the pressures. Upon termination of the gradient, diluent buffer was flowed exclusively for 15 min at 30 μL/min as a washout or sensor dissociation step. Spectra were recorded every 7 s for all output channels containing two rings (capture and negative control probe) and were exported as.omr files for subsequent data processing.

### Data Processing

Data files (.omr) of raw spectra from a single experiment were uploaded to a previously described Python-based script, allowing resonance wavelengths for each 6 nm sweep (1547 to 1553 nm) to be extracted.^31^ These peak positions were stored in a single PARQUET file, then plotted as a function of time in three formats: relative capture and control ring shifts, subtracted relative shifts, and raw wavelength shifts. Relative shifts refer to values after subtraction of the baseline (t = 0) shift. The resulting relative and subtracted relative shifts were saved as .csv files for each output channel and could be further analyzed by conversion to a .xlsx file.

### Data Analysis and Fitting

**Figure 3:**
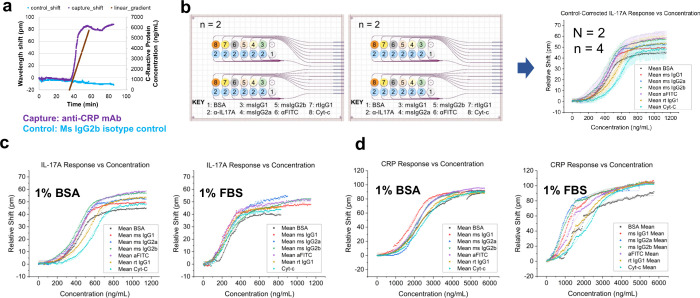
Replicates and calibration of the analyte gradient sensor response for IL-17A and CRP. (a) Representative output sensorgram showing redshift vs time for the capture and control sensors, along with the nominal CRP concentration plotted on the secondary *y*-axis in BSA diluent. (b) Schematic showing biological replicates per chip as redundant ring banks functionalized identically (n), and technical replicates (N) shown as multiple chips used for each response calibration. (c) Calibration curves generated for IL-17A in both 1% BSA and 1% FBS background with each reference panel subtracted. (d) Calibration curves generated for CRP in both 1% BSA and 1% FBS background with each reference panel subtracted.

Subtracted relative shifts for the entire assay were output to .xlsx files and plotted for further analysis. For analysis of the linear gradient region, the timestamps of .omr files were noted and correlated to the recorded gradient start time. This allowed zeroing at t = 0 of the subtracted relative shifts unique to the gradient region and ensured consistency between the time courses across different output channels and assays. Thus, identical start and stop times were identified and used for global analysis of analyte detection for both IL-17A and CRP. The resulting data were evaluated based on four major criteria: assay linearity, accuracy (recovery), precision (reproducibility), and selectivity. Before these metrics could be properly assessed, response standard curves were generated for IL-17A and CRP in both nonserum (BSA) and serum backgrounds (Figure 3b-d). The standards were fit with a four-parameter logistic (4PL) model using OriginPro (Originlab, Inc.) and the resulting equations were rearranged to solve for concentration. Given the relative shifts from our experimental (gradient) assays, interpolating the standard curves provided a measurement of concentration. To evaluate assay linearity, the expected IL-17A and CRP concentrations were plotted against the measured concentrations using each negative control (reference) probe. Linear fits to these data revealed the slopes and goodness-of-fit (R^2^). Accuracy, or analytical recovery, was determined by repeating assays with the same conditions as the calibration experiments and measuring the resultant concentrations based on the shifts observed (interpolation of the standard curves). The difference between the actual gradient concentrations and measured gradient concentrations was assessed using the percentage difference equation (eq 1):
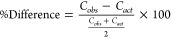
1

If the percent difference was calculated to be 20%, for example, the analytical recovery was reported as 80%. This condition was set as the acceptability threshold for accuracy. We required that at least 65% of the experimental assay data points in the control-subtracted response met this threshold. With respect to precision, the coefficients of variation (CV) for interpolated concentrations on the same PIC were calculated (intra-assay), and across different PICs (inter-assay). The duplication of ring banks on a single PIC allowed for the intra-assay CV values to be calculated, comparing variability between two identically functionalized sensors in a single assay. The acceptability metric for CV was set at 20% with at least 65% of the data set required to conform to this threshold. The 65% acceptance criterion was inspired by the *FDA Bioanalytical Method Validation* guidance that at least 67% of QC data points should fall within 20% of their nominal value.^37^ Lastly, selectivity was determined based on the lower limit of quantitation (LLOQ). Before LLOQ could be calculated, it was necessary to determine the limit of detection (LOD) for each control-subtracted response across all assays. LOD was calculated as LOD = LOB + 1.645σ_low conc_, where LOB = μ_blank_ + 1.645σ_blank_; μ_blank_ represents the mean values of the subtracted relative shifts in the absence of analyte and σ_low conc_ is the standard deviation of the response during the first minute of the gradient assay.^38−40^ The LOD and LOB were obtained using a method described by Armbruster and Pry.^41^ Since LLOQ ≥ LOD in any biological assay, the first data point that yielded a concentration fulfilling this condition and demonstrating a CV of ≤20% was selected as the LLOQ. This was performed mainly as an interassay analysis since multiple data sets were needed to achieve statistical confidence and meaningful parameters such as mean and standard deviation. Selectivity for the IL-17A and CRP negative-control subtracted detection was met when the LLOQ in serum background, i.e. 1% FBS in PBS-T, was equal to or less than the LLOQ in a nonserum background, i.e. 1% BSA in PBS-T.

### Scoring of Control Performance

A scoring system was developed to objectively assign points to each of the following categories: linearity, R-squared, accuracy, intra-CV, inter-CV, and selectivity. For each category, the points possible were equal to the number of runs that were available to evaluate, and the points awarded were equal to the number of runs that met the predetermined criteria for a metric. Fields that are blank did not have any available data to score for that category, due to failure of that sensor channel. Points were tallied across all categories for each of the negative control probes, then divided by the total number of possible points, generating a grade out of 1.0. The negative-control subtracted detection for IL-17A and CRP with the highest-grade score was defined as the most robust performer in terms of correcting for nonspecific binding and analytical reliability.

## Results

Target analytes were studied in a 1% BSA buffer, endothelial growth medium (EGM-2) containing 1% BSA, and 1% fetal bovine serum (FBS). Note that for the CRP test case, 1 and 20% FBS conditions were used. These assay backgrounds were chosen to represent diverse sensor applications, and to determine if matrix effects caused differences in assay performance.^7,42^ Supplemented EGM-2 contains insulin, growth factors, and BSA, while FBS contains numerous endogenous proteins that will nonspecifically bind to other macromolecules and the PIC surface.^43,44^ To measure binding, we adapted a previously described microfluidic gradient generator assay to our instrumentation. In this method, linearly increasing concentrations of analyte were flowed over the sensor surface as a function of time (Figure 2).^45^ Assay performance of each negative control-subtracted response was assessed based on linearity, accuracy (recovery), precision (reproducibility), and selectivity. These metrics are widely used in validations of FDA-regulated bioanalytical methods and were implemented as guidelines in this work with some flexibility in the thresholds.^37^

Initial validation of the gradient generator using bovine serum albumin (BSA) as the test analyte demonstrated a linear concentration profile as a function of time across three independent runs (Figure 2d). The average slope was found to be 0.452 ± 0.01 mg/mL·min with R^2^ > 0.993. Following the addition of a microfluidic valve to reduce the likelihood of bubbles that could disrupt optical measurements, performance was verified by repeating the experiment testing the linearity of BSA concentrations. The resulting fit demonstrated a slope of 0.456 mg/mL·min with R^2^ = 0.998, similar to previous parameters. The linear fits of BSA concentration versus time served as a model for the rate of change of analyte concentration, and allowed calculation of the expected gradient concentration of any analyte at a given time, since the starting concentration was known. For IL-17A and CRP, the starting concentrations in the analyte reservoir were 1 μg/mL and 5 μg/mL, respectively. A proportion was set up to solve for the characteristic slopes of each linear analyte gradient (Supporting Information). Because both pumps of the gradient generator were controlled by PC software, an algorithm was developed to automate the analyte-buffer mixing process and subsequent gradient formation through modulating the flow rates and pressures. Opposing linear flow rate ramps were required to achieve the desired output concentrations.

Next, replicate linear gradient assays were conducted with IL-17A and CRP in protein and low-serum backgrounds (Figure 3a, b). Each output channel of the PhRR PIC provided an independent, nonsubtracted assay response of both the capture antibody and reference probe sensors (Figure 3a). The averaged, reference-subtracted IL-17A and CRP relative shifts were calculated for each negative control probe and plotted as a function of concentration (Figure 3c, d). In general, the maximum responses for IL-17A were consistent across different backgrounds, while the maximum responses and EC_50_ values (inflection points) for CRP were more variable in 1% FBS than in 1% BSA. All data were fit with a four-parameter logistic (4PL) equation, which approximates the binding behavior of an immobilized bioreceptor plus analyte. Error (not plotted in Figure 3) was determined by propagating the intra- and inter-PIC standard deviations by calculating the square root of the sum of squares. As expected for a refractive index biosensor which operates on mass capture, the maximum response (relative shift) of the CRP standard at saturation is markedly higher than that of the IL-17A standard, due to the higher molecular weight of CRP.

**Figure 4:**
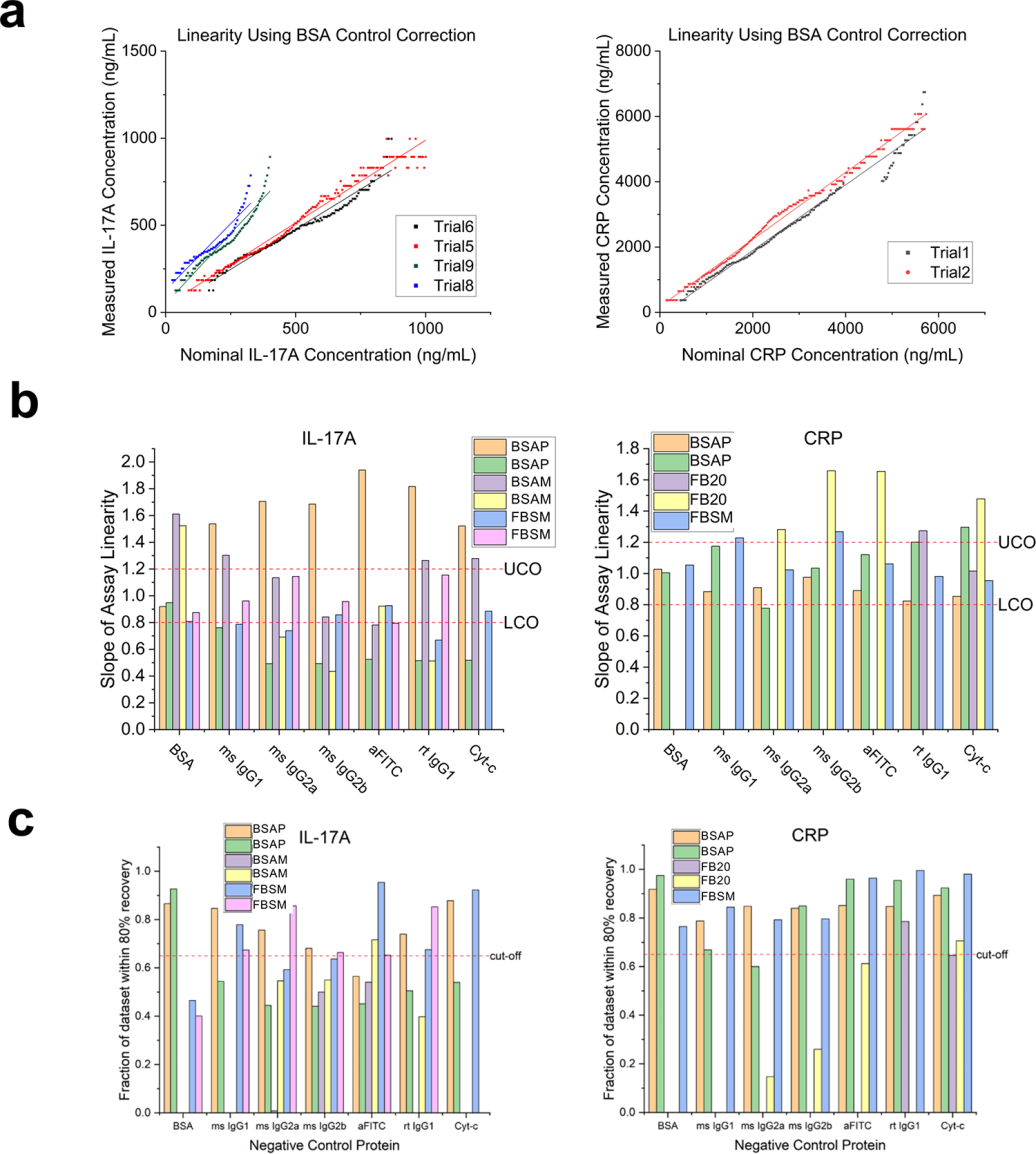
Parameters used for the evaluation of assay performance and assessment of reference control subtraction (a) Example of assay linearity evaluation using BSA as the reference control for both IL-17A (left; two trials in each of two different media formats) and CRP (right). The measured analyte concentration was plotted against the expected (nominal) analyte concentration and these data were fit with a linear model (*y* = *mx* + *b*) and the slope, m, and goodness of fit, *R*^2^, were obtained for several trials in 1% BSA background. (b) Bar graphs showing slope as a function of negative control protein for several trials in both BSA and serum backgrounds for IL-17A (left) and CRP (right) with the indicated acceptability cutoffs (red dashed line). (c) Bar graphs showing the fraction of data points that fell within the 80% recovery (or 20% difference) threshold as a function of negative control protein for several trials in both BSA and serum backgrounds for IL-17A (left) and CRP (right) with the indicated acceptability cutoff of 0.65 (red dashed line). Abbreviations: BSAP: 1% BSA in PBS-T; BSAM: 1% BSA in EGM-2; FBSM: 1% FBS in EGM-2; FB20:20% FBS in AWB (PBS-ET).

Using established FDA guidelines, we formulated acceptability criteria to evaluate biosensor performance contingent on the negative control probe in question.^37^ First, assay linearity was assessed across all controls and background conditions for both analytes. Here, the measured analyte concentration (based on interpolation of the calibrations in Figure 3) was plotted against the nominal (expected) analyte concentration (eqs 3 and 4; Supporting Information). A slope of unity signifies a 1:1 input and output relationship, the desired condition for bioanalytical assays. Of all reference controls, BSA-subtracted detection had the highest proportion of assays that fell within the slope criterion of 1 ± 0.2 for both IL-17A and CRP linearity (Figure 4b, Supplementary Figures S4 – S7). Mouse IgG2b isotype control was second for IL-17A, while anti-FITC and rat IgG1 isotype control were equally second-best for the CRP case. Apart from rat IgG1-subtracted IL-17A detection, all other reference controls provided R^2^ values for assay linearity above the threshold of 0.8 (Figure S8). A possible explanation for the lower R^2^ of the rat IgG1-subtracted IL-17A data is the observed curvature in the assay linearity plot, where an inflection point is observed around 500 ng/mL of nominal concentration (Figure S6).

The accuracy, or analytical recovery, of IL-17A and CRP detection with reference control subtraction was determined by repeating the linear gradient assays in BSA and FBS background (with and without cell culture medium) and comparing these to standard curves described above. The percentage difference in analyte concentration was calculated using eq 1. To be considered an acceptable recovery, the % difference between the expected and the measured analyte concentration was not to exceed 20% for *at least 65%* of the data set for a single linear gradient assay (Table 1). Mouse IgG1 isotype control-subtracted IL-17A recovery showed the most assays conforming to the cutoff, while rat IgG1 isotype control-subtracted CRP recovery had the most assays falling within the acceptability metric (Figure 4c). For IL-17A, rat IgG1 isotype control and cytochrome c negative control probes also scored favorably in terms of accuracy, while all negative control probes except for mouse IgG2a isotype control scored well for CRP reference subtraction. In these cases, the performance was not affected by the type of background matrix (serum-free vs serum-rich). Of interest is that the slope of the assay linearity for each control probe depended on the sample matrix: for example, responses for IL-17A using BSA as the control were reproducible in each serum-free matrix, but differed significantly for BSAP vs BSAM (Figure 4A). There are also differences in assay linearity reproducibility (for example performance of IL-17A response in Figure 4B). These are considered as part of our cutoffs for this metric.

**Table 1:** Acceptability Criteria for Linearity, Accuracy, Precision, and Selectivity Metrics Obtained from Assays

**metric**	**acceptability criteria**
linearity	R2 ≥ 0.8; slope = 1 ± 0.2
accuracy (recovery)	percent difference within ±20% of nominal value for at least 65% of the data set:
precision (reproducibility)	coefficient of variation (CV) ± 20% for at least 65% of the data set
selectivity	LLOQ within ±20% between serum and nonserum backgrounds

**Figure 5:**
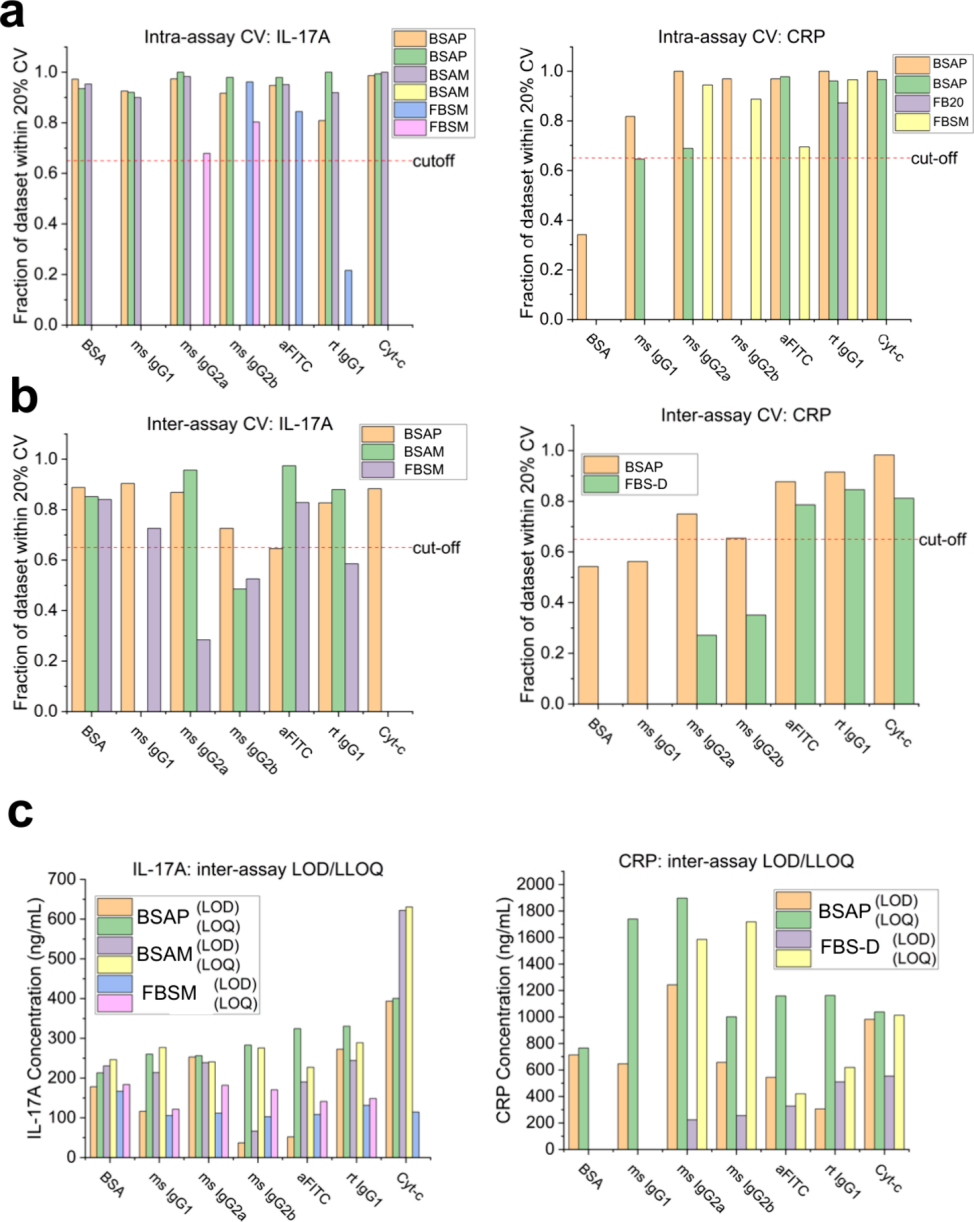
Assay precision and selectivity. (a) Fraction of intra-assay data points that are within 20% CV for trials in BSA and serum backgrounds for IL-17A (left) and CRP (right) as a function of negative control subtraction. The cutoff of 0.65 is indicated with a red dashed line. (b) Fraction of interassay data points that are within 20% CV for trials in BSA and serum backgrounds for IL-17A (left) and CRP (right) as a function of negative control subtraction. The cutoff of 0.65 is indicated with a red dashed line. (c) Measured concentrations corresponding to the limits of detection (LOD) and lower limit of quantitation (LLOQ) as a function of negative control protein for both IL-17A and CRP across several trials conducted in either 1% BSA or serum. LOD = LOB + 1.645σ. Abbreviations: FBS-D: FBS-containing diluent.

Next, we evaluated the precision (reproducibility) with respect to reference control subtraction. We established that the coefficient of variation (CV) was not to exceed 20% for *at least 65%* of the data points within an assay (intra-CV), which compares two ring resonator sensors on the same PIC, and across assays (inter-CV), comparing sensors on different PICs. All reference controls resulted in IL-17A detection that was well within the intra-CV acceptability criterion; only rat IgG1 isotype control demonstrated one assay that fell outside the cutoff metric (Figure 5a). Reference control subtraction for CRP showed that intra-CV was the tightest for rat IgG1 isotype control, anti-FITC, and mouse IgG2a isotype control with mouse IgG2b isotype control and cytochrome c also performing adequately. For inter-CV evaluation, BSA subtraction resulted in the highest proportion of IL-17 assays falling within the 20% cutoff, while all assays using the anti-FITC, rat IgG1 isotype control, and cytochrome c subtraction fell within the established CV metrics for CRP detection (Figure 5b). The precision of each negative control-subtracted IL-17A and CRP response did not appear to be influenced by the type of assay background used.

Selectivity is the ability of an assay to detect and measure the target analyte in the presence of potential interferents and other constituents such as serum proteins.^46^ It was critical to test how the reference control modulated selectivity in both the IL-17A and CRP assays to ascertain the true binding response in the presence of abundant serum proteins. To accomplish this, we calculated the lower limits of quantitation (LLOQ) for each reference control in both BSA and FBS backgrounds. Our acceptability criterion stated that the LLOQ in serum backgrounds must be less than or equal to the LLOQ in nonserum backgrounds. In other words, the LLOQ was not to deteriorate in serum-rich assay backgrounds. For IL-17A, all reference controls showed improved LLOQ in 1% FBS backgrounds. Mouse IgG1 isotype control yielded the lowest IL-17A LLOQ, nearing 121.4 ng/mL (Figure 5c). Cytochrome c-corrected LLOQ in serum could not be determined due to a defect in the output channel of the PIC. For CRP, the control-subtracted detection showed that mouse IgG2a isotype control, anti-FITC, rat IgG1 isotype control, and cytochrome c all improved LLOQ in assays conducted in 1% FBS backgrounds relative to serum-free. Anti-FITC corrected CRP detection showed the lowest LLOQ nearing 420.7 ng/mL (Figure 5c).

**Figure 6:**
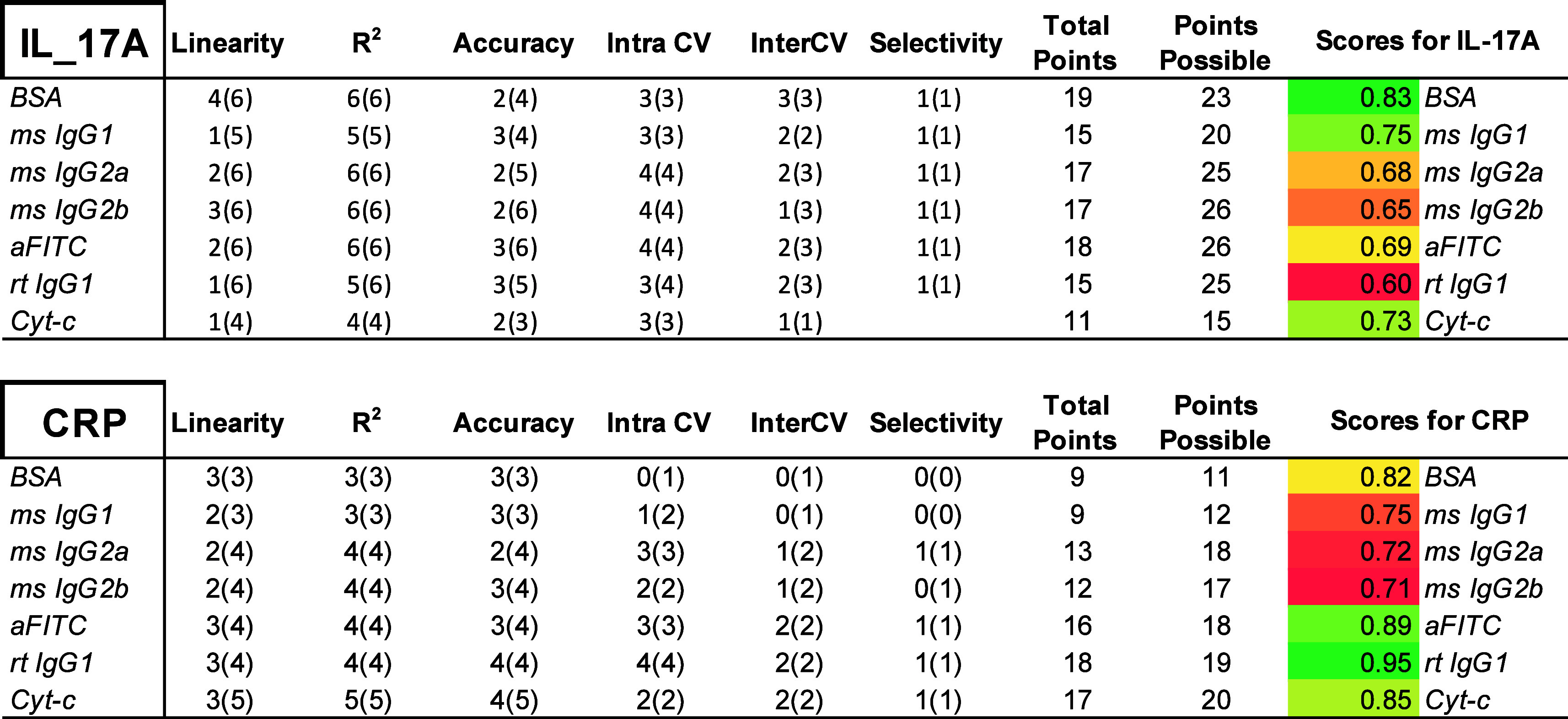
Breakdown of scoring criteria for each negative control probe and summary table indicating the top performers (a) BSA reference (negative control) probe scored the highest for assay performance metrics and gave the most reliable IL-17A detection data. Each metric is color-coded and graded based on total possible points overall. The ratio of points scored/points possible yielded the final grade out of 1.0. (b) Rt IgG1 isotype reference (negative control) probe scored the highest for assay performance metrics and gave the most reliable CRP detection data.

To encapsulate all findings, a scoring (point) system was developed to determine the most reliable and robust reference control for IL-17A and CRP detection. All metrics were divided into categories and assigned a possible point number corresponding to the number of assays conducted (Figure 6). Points were assigned based on successful fulfillment of criteria for: linearity, R-squared (goodness of fit), accuracy (recovery), intrachip CV, interchip CV, and selectivity. Total points were tallied across all categories and matrices and divided by the total *possible* number of points to generate a reliability score out of 1.0. BSA (0.83), mouse IgG1 isotype control (0.75), and cytochrome c (0.73) scored the highest for IL-17A, while rat IgG1 isotype control (0.95), anti-FITC (0.89), and cytochrome c (0.85) provided the highest scores for CRP detection.

## Discussion

We have developed a template for identifying the most appropriate negative control probe in a biosensor assay, by screening a 7-plex array of negative control probes (on a photonic biosensor chip) and testing their ability to correct for nonspecific binding of the paired capture antibody probes as well as small fluctuations in the refractive index of the sensing medium. These studies were facilitated by a linear gradient generator. Thus, analyte responses were calibrated on a single sensor chip in less than an hour for 7 different reference controls and two monoclonal capture antibodies. Others in the field have shown the utility of analyte gradient generators for *in vitro* drug screening, determination of kinetic and thermodynamic binding parameters, and perfusion of cellular monolayers.^45,47−49^ To our knowledge, this is the first example of an optical biosensor using a linear analyte gradient generator to interrogate a panel of nonspecific binding control probes to establish specificity. These results suggest that, perhaps, isotype matching of the capture antibody is not always optimal for subtraction of the nonspecific binding response.

We observed that the response calibration plots were adequately modeled with 4PL fits regardless of assay background (serum vs nonserum) and that the saturation regions were consistent for both IL-17A and CRP. Linearity, accuracy (percent recovery), and precision (reproducibility) were robust for several controls. One potential limitation of this analysis can be observed in Figures S5 and S6 where the assay plots deviate from linearity at the midpoint of the concentration sweep. We posit that this reduction in assay linearity occurs toward higher concentrations due to a transition in the sigmoidal response-concentration curve from the linear region to the upper asymptotic region. This observation reinforced the need to include assay linearity as a criterion for negative control selection.

While one could envision various ways to evaluate the results of these assays, we chose to score each negative control probe candidate for meeting success criteria in accuracy (percent recovery), precision (intra- and interassay CV), and selectivity (retaining equal or improved LOQ) in the presence of serum background. The fact that BSA control subtraction scored the best for IL-17A is somewhat unexpected as it is less than half the molecular weight of the capture molecule to IL-17A, an IgG. Although the biophysical properties on the sensor surface such as steric crowding are anticipated to be different between BSA and IgG, both are globular proteins and BSA is present in the running buffer of the assay. The initial hypothesis that isotype matching of the negative control probe to the capture antibody would most adequately subtract nonspecific binding appeared to be a reasonable prediction as mouse IgG1 scored as the second-best negative control for IL-17A, which is a mouse IgG1 itself. A parallel argument was made for CRP as the capture antibody used in this study was a mouse IgG2b isotype. Perhaps more surprisingly, rat IgG1 isotype scored the highest for CRP while mouse IgG2b scored the poorest. The amino acid sequence identity between mouse IgG2b and rat IgG1 is low (Supplementary Figure S9), suggesting that mouse IgG2b and rat IgG1 are unlikely to correct for nonspecific binding in the same way. This is particularly evident when comparing the hydropathy index of the core hinge position of the IgG molecule where mouse IgG2b is −0.75 and rat IgG1 is 1.1. The discrepancy in the isotype-matched mouse IgG2b and rat IgG1 control correction for CRP *could* be explained by the biochemical nature of the molecule (hydrophobicity) along with the isoelectric point of the protein. A closer look into the isoelectric point of the CRP analyte, capture antibody, mouse IgG2b, and rat IgG1 isotype control probe would reveal whether electrostatic interactions are likely to disrupt or favor binding interactions. One caveat is that this method omits the effects of hydrogen bonding and van der Waals interactions. Although potentially informative, such an analysis is outside the scope of this work.

## Conclusions

To our knowledge, this work is the first example of a systematic analysis of negative control selection for biosensors with the purpose of subtracting the nonspecific binding signal. We tested 7 negative control probes simultaneously given either anti-IL-17A or anti-CRP capture antibody pairings to determine which reference control protein most faithfully and consistently removed nonreal analyte capture due to nonspecific interaction. Our analysis of assay metrics, inspired by FDA guidelines, suggested that there is a dependence on the capture antibody and analyte in determining the most appropriate reference control. While the differences in assay performance for IL-17A and CRP were found to be subtle, the best-scoring reference control based on the bioanalytical parameters of linearity, accuracy, and selectivity differed for each analyte. In the IL-17A assay, BSA scored the highest at 83%, while mouse IgG1 isotype control antibody placed a close-second with 75%. With respect to the CRP assay, the rat IgG1 isotype control antibody scored the highest at 95%, while anti-FITC scored the second highest at 89%. Quantitative control probe-dependent differences in assay metrics observed with these example analytes highlight the need for similar studies to be done with each analyte to be studied. An issue with the data that might be raised as a potentially confounding factor is that different control probes likely react with the surface-bound epoxide with different efficiencies, and thus the amount of each control probe immobilized may not be equal despite similar spotting concentrations. However, as this is a systematic rather than a random error (amount immobilized for each control may vary within chip, but will be identical chip to chip) it does not reduce the validity of the method.

Our approach highlights a key advantage of multiplex sensors that has rarely (if ever) been noted: in addition to enabling detection of multiple *analytes* simultaneously, such sensors also enable simultaneous calibration of analytes against multiplex *control probes*. For singleplex sensors such as most commercially available surface plasmon resonance (SPR) instruments, identification of the best-performing control probe would incur significant time and material costs.

It is axiomatic that comparison of the “specific” binding signal against a “nonspecific” (control) binding signal using a reference set of samples and simultaneous measurements of multiple controls will allow determination of the analyte/control pair providing the best analytical performance. As such, the methodology reported here will prove useful for other multiplex label-free sensors, regardless of detection method or surface type. Of course, other negative control probes could also be incorporated into the analysis. Candidates include aprotinin, a strongly basic protein, and IgG isotypes from other species. We also anticipate that parameters of the linear gradient generator, such as flow rate, can be tuned to span a wider concentration range, and presumably increase the amount of usable calibration response data. Studies to those ends are in progress in our laboratory, as well as testing the approach described here on other biomarker proteins of interest.

## Supporting Information Available

Extended methods, analysis of assay linearity, and analysis of control probes by hydropathy index (PDF)

## References

[ref1] Bănică F.-G. What are Chemical Sensors? In Chemical Sensors and Biosensors: Fundamentals and Applications; John Wiley & Sons, 2012; pp 1–20.

[ref2] Vigneshvar S.; Sudhakumari C. C.; Senthilkumaran B.; Prakash H. Recent advances in biosensor technology for potential applications - an overview. Front. Bioeng. Biotechnol. 2016, 4, 11 10.3389/fbioe.2016.00011. 26909346 PMC4754454

[ref3] Kim J.; Campbell A. S.; de Ávila B. E. F.; Wang J. Wearable biosensors for healthcare monitoring. Nat. Biotechnol. 2019, 37, 389–406. 10.1038/s41587-019-0045-y. 30804534 PMC8183422

[ref4] Zhang H.; Yu D.; Zhao Y.; Fan A. Turn-on chemiluminescent sensing platform for label-free protease detection using streptavidin-modified magnetic beads. Biosens. Bioelectron. 2014, 61, 45–50. 10.1016/j.bios.2014.04.050. 24846776

[ref5] Burry R. W. Controls for immunocytochemistry: An update. J. Histochem. Cytochem. 2011, 59, 6–12. 10.1369/jhc.2010.956920. 20852036 PMC3201116

[ref6] Makowski E. K.; Wu L.; Desai A. A.; Tessier P. M. Highly sensitive detection of antibody nonspecific interactions using flow cytometry. MAbs 2021, 13, 1951426 10.1080/19420862.2021.1951426. 34313552 PMC8317921

[ref7] Frutiger A.; Tanno A.; Hwu S.; Tiefenauer R. F.; Vörös J.; Nakatsuka N. Nonspecific Binding - Fundamental Concepts and Consequences for Biosensing Applications. Chem. Rev. 2021, 121, 8095–8160. 10.1021/acs.chemrev.1c00044. 34105942

[ref8] Myszka D. G. Improving biosensor analysis. J. Mol. Recogn. 1999, 12, 279–284. 10.1002/(SICI)1099-1352(199909/10)12:5<279::AID-JMR473>3.0.CO;2-3. 10556875

[ref9] Arney J. W.; Weeks K. M. RNA-Ligand Interactions Quantified by Surface Plasmon Resonance with Reference Subtraction. Biochemistry 2022, 61, 1625–1632. 10.1021/acs.biochem.2c00177. 35802500 PMC9357220

[ref10] Bello V.; Vandezande W.; Daems D.; Lammertyn J. V. Design and Implementation of a Dual-Region Self-Referencing Fiber-Optic Surface Plasmon Resonance Biosensor. ACS Sens. 2022, 7, 3360–3368. 10.1021/acssensors.2c01362. 36269596

[ref11] Eddings M. A.; Eckman J. W.; Arana C. A.; Papalia G. A.; Connolly J. E.; Gale B. K.; Myszka D. G. ‘Spot and hop’: Internal referencing for surface plasmon resonance imaging using a three-dimensional microfluidic flow cell array. Anal. Biochem. 2009, 385, 309–313. 10.1016/j.ab.2008.11.014. 19059374 PMC6691735

[ref12] Kwong Hong Tsang D.; Lieberthal T. J.; Watts C.; Dunlop I. E.; Ramadan S.; del Rio Hernandez A. E.; Klein N. Chemically Functionalised Graphene FET Biosensor for the Label-free Sensing of Exosomes. Sci. Rep. 2019, 9, 13946 10.1038/s41598-019-50412-9. 31558796 PMC6763426

[ref13] Barton A. C.; Collyer S. D.; Davis F.; Garifallou G.-Z.; Tsekenis G.; Tully E.; O’Kennedy R.; Gibson T.; Millner P. A.; Higson S. P. J. Labeless AC impedimetric antibody-based sensors with pg mL-1 sensitivities for point-of-care biomedical applications. Biosens, Bioelectron. 2009, 24, 1090–1095. 10.1016/j.bios.2008.06.001. 18653325

[ref14] Horiuchi T.; Miura T.; Iwasaki Y.; Seyama M.; Inoue S.; Takahashi J.-I.; Haga T.; Tamechika E. Passive fluidic chip composed of integrated vertical capillary tubes developed for on-site SPR immunoassay analysis targeting real samples. Sensors 2012, 12, 7095–7108. 10.3390/s120607095. 22969339 PMC3435968

[ref15] Brown H. M. G.; Kuhns M. M.; Maxwell Z.; Arriaga E. A. Nonspecific binding correction for single-cell mass cytometric analysis of autophagy and myoblast differentiation. Anal. Chem. 2021, 93, 1401–1408. 10.1021/acs.analchem.0c03211. 33348978 PMC9487344

[ref16] Luan E.; Shoman H.; Ratner D. M.; Cheung K. C.; Chrostowski L. Silicon photonic biosensors using label-free detection. Sensors 2018, 18, 3519 10.3390/s18103519. 30340405 PMC6210424

[ref17] Bogaerts W.; De Heyn P.; Van Vaerenbergh T.; De Vos K.; Kumar Selvaraja S.; Claes T.; Dumon P.; Bienstman P.; Van Thourhout D.; Baets R. Silicon microring resonators. Laser Photon. Rev. 2012, 6, 47–73. 10.1002/lpor.201100017.

[ref18] Sarkaleh A.; Lahijani B.; Saberkari H.; Esmaeeli A. Optical Ring Resonators: A Platform for Biological Sensing Applications. J. Med. Signals Sens. 2017, 7, 185–191. 10.4103/jmss.JMSS_9_17. 28840120 PMC5551303

[ref19] Nanophotonic waveguides in silicon-on-insulator fabricated with CMOS technology. J. Lightwave Techno. 2005, 23, 401-412 10.1109/JLT.2004.834471.

[ref20] Carlborg C. F.; Gylfason K. B.; Kaźmierczak A.; Dortu F.; Bañuls Polo M. J.; Maquieira Catala A.; Kresbach G. M.; Sohlström H.; Moh T.; Vivien L.; Popplewell J.; Ronan G.; Barrios C. A.; Stemme G.; van der Wijngaart W. A packaged optical slot-waveguide ring resonator sensor array for multiplex label-free assays in labs-on-chips. Lab Chip 2010, 10, 281–290. 10.1039/B914183A. 20090999

[ref21] Castelló-Pedrero L.; Gómez-Gómez M. I.; García-Rupérez J.; Griol A.; Martínez A. Performance improvement of a silicon nitride ring resonator biosensor operated in the TM mode at 1310 nm. Biomed. Opt. Express 2021, 12, 7244–7260. 10.1364/BOE.437823. 34858712 PMC8606153

[ref22] Millar N. L.; Akbar M.; Campbell A. L.; Reilly J. H.; Kerr S. C.; McLean M.; Frleta-Gilchrist M.; Fazzi U. G.; Leach W. J.; Rooney B. P.; Crowe L. A. N.; Murrell G. A. C.; McInnes I. B. IL-17A mediates inflammatory and tissue remodelling events in early human tendinopathy. Sci. Rep. 2016, 6, 27149 10.1038/srep27149. 27263531 PMC4893609

[ref23] Liu Z.; Huang F.; Luo G.; Wang Y.; Du R.; Sun W.; Li J.; Yuan X.; Cao D.; Li Y.; Liu C.; Liang S.; Jin X.; Ling S.; Wang D.; Li Y. miR-214 stimulated by IL-17A regulates bone loss in patients with ankylosing spondylitis. Rheumatology 2020, 59, 1159–1169. 10.1093/rheumatology/kez594. 31846044

[ref24] Pathak A.; Agrawal A. Evoluation of C-reactive protein. Front. Immunol. 2019, 10, 943 10.3389/fimmu.2019.00943. 31114584 PMC6503050

[ref25] Jiang X.; Zhang C.; Pan Y.; Cheng X.; Zhang W. Effects of C-reactive protein trajectories of critically ill patients with sepsis on in-hospital mortality rate. Sci. Rep. 2023, 13, 15223 10.1038/s41598-023-42352-2. 37709919 PMC10502021

[ref26] McGovern J.; Wadsworth J.; Catchpole A.; Richards C.; McMillan D. C.; Kelliher T.; Goodall E.; Murray E.; Melaugh T.; McPhillips S.; Brice K.; Barbour K.; Robinson S.; Moffitt P.; Kemp O.; Talwar D.; Maguire D. The relationship between micronutrient status, frailty, systemic inflammation, and clinical outcomes in patients admitted to hospital with COVID-19. J. Transl. Med. 2023, 21, 284 10.1186/s12967-023-04138-y. 37118813 PMC10139911

[ref27] Cognetti J. S.; Steiner D. J.; Abedin M.; Bryan M. R.; Shanahan C.; Tokranova N.; Young E.; Klose A. M.; Zavriyev A.; Judy N.; Piorek B.; Meinhart C.; Jakubowicz R.; Warren H.; Cady N. C.; Miller B. L. Disposable photonics for cost-effective clinical bioassays: application to COVID-19 antibody testing. Lab Chip 2021, 21, 2913–2921. 10.1039/D1LC00369K. 34160511

[ref28] Steiner D. J.; Cognetti J. S.; Luta E. P.; Klose A. M.; Bucukovski J.; Bryan M. R.; Schmuke J. J.; Nguyen-Contant P.; Sangster M. Y.; Topham D. J.; Miller B. L. Array-based analysis of SARS-CoV-2, other coronaviruses, and influenza antibodies in convalescent COVID-19 patients *Biosens*. Bioelectron. 2020, 169, 112643 10.1016/j.bios.2020.112643. PMC7522665 33007615

[ref29] Cognetti J. S.; Miller B. L. Monitoring serum spike protein with disposable photonic biosensors following SARS-CoV-2 vaccination. Sensors 2021, 21, 5857 10.3390/s21175857. 34502753 PMC8434114

[ref30] Zhang H.; Klose A. M.; Miller B. L. A label-free, multiplex glycan microarray biosensor for influenza virus detection. Bioconj. Chem. 2021, 32, 533–540. 10.1021/acs.bioconjchem.0c00718. 33559468

[ref31] Cognetti J. S.; Moen M. T.; Brewer M. G.; Bryan M. R.; Tice J. D.; McGrath J. L.; Miller B. L. A photonic biosensor-integrated tissue chip platform for real-time sensing of lung epithelial inflammatory markers. Lab Chip 2023, 23, 239–250. 10.1039/D2LC00864E. 36594179 PMC10311125

[ref32] Qavi A. J.; Meserve K.; Aman J. M.; Vu H.; Zeitlin L.; Dye J. M.; Froude J. W.; Leung D. W.; Yang L.; Holtsberg F. W.; Bailey R. C.; Amarasinghe G. K. Rapid detection of an Ebola biomarker with optical microring resonators. Cell Rep. Meth. 2022, 2, 100234 10.1016/j.crmeth.2022.100234. PMC9243524 35784644

[ref33] Meserve K.; Qavi A. J.; Aman M. J.; Vu H.; Zeitlin L.; Dye J. M.; Froude J. W.; Leung D. W.; Yang L.; Holtsberg F. W.; Amarasinghe G. K.; Bailey R. C. Detection of biomarkers for filoviral infection with a silicon photonic resonator platform. STAR Protoc 2022, 3, 101719 10.1016/j.xpro.2022.101719. 36153732 PMC9515683

[ref34] Li Z.; Zou J.; Zhu H.; Nguyen B. T. T.; Shi Y.; Liu P. Y.; Bailey R. C.; Zhou J.; Wang H.; Yang Z.; Jin Y.; Yap P. H.; Cai H.; Hao Y.; Liu A. Q. Biotoxoid Photonic Sensors with Temperature Insensitivity Using a Cascade of Ring Resonator and Mach-Zehnder Interferometer. ACS Sens. 2020, 5, 2448–2456. 10.1021/acssensors.0c00622. 32666782

[ref35] Valera E.; Shia W. W.; Bailey R. C. Development and validation of an immunosensor for monocyte chemotactic protein 1 using a silicon photonic microring resonator biosensing platform. Clin. Biochem. 2016, 49, 121–126. 10.1016/j.clinbiochem.2015.09.001. 26365696 PMC4715927

[ref36] Valera E.; McClellan M. S.; Bailey R. C. Magnetically-actuated, bead-enhanced silicon photonic immunosensor. Anal. Meth. 2015, 7, 8539–8544. 10.1039/C5AY01477H. PMC4627713 26528374

[ref37] U.S. Department of Health and Human Services Food and Drug Administration Center for Drug Evaluation and Research (CDER) Center for Veterinary Medicine (CVM) Bioanalytical Method Validation Guidance for Industry 2018. https://www.fda.gov/regulatory-information/search-fda-guidance-documents/bioanalytical-method-validation-guidance-industry (Accessed: Mar. 28, 2023).

[ref38] Barry J. A.; Groseclose M. R.; Castellino S. Quantification and assessment of detection capability in imaging mass spectrometry using a revised mimetic tissue model. Bioanalysis 2019, 11, 1099–1116. 10.4155/bio-2019-0035. 31251106

[ref39] Duan Y.; Wu W.; Zhao Q.; Liu S.; Liu H.; Huang M.; Wang T.; Liang M.; Wang Z. Enzyme-antibody-modified gold nanoparticle probes for the ultrasensitive detection of nucleocapsid protein in SFTSV. Ing. J. Environ. Res. Pub. Health 2020, 17, 4427 10.3390/ijerph17124427. PMC7344430 32575570

[ref40] Garrido E.; Climent E.; Marcos M. D.; Sancenón F.; Rurack K.; Martínez-Máñez R. Dualplex lateral flow assay for simultaneous scopolamine and ‘cannibal drug’ detection based on receptor-gated mesoporous nanoparticles. Nanoscale 2022, 14, 13505–13513. 10.1039/D2NR03325A. 36102017

[ref41] Armbruster D. A.; Pry T. Limit of blank, limit of detection and limit of quantitation. Clin. BIochem. Rev. 2008, 29, S49–S52. 18852857 PMC2556583

[ref42] Lane J. S.; Richens J. L.; Vere K. A.; O’Shea P. Rational targeting of subclasses of intermolecular interactions: Elimination of nonspecific binding for analyte sensing. Langmuir 2014, 30, 9457–9465. 10.1021/la5016548. 25046104

[ref43] Liu S.; Yang W.; Li Y.; Sun C. Fetal bovine serum, an important factor affecting the reproducibility of cell experiments. Sci. Rep. 2023, 13, 1942 10.1038/s41598-023-29060-7. 36732616 PMC9894865

[ref44] Eggert S.; Wiest J.; Rosolowski J.; Weber T. Practical Workshop on Replacing Fetal Bovine Serum (FBS) in Life Science Research: From Theory into Practice. ALTEX 2022, 39, 712–713. 10.14573/altex.2207071. 36317781

[ref45] Marty M. T.; Sloan C. D. K.; Bailey R. C.; Sligar S. G. Nonlinear analyte concentration gradients for one-step kinetic analysis employing optical microring resonators. Anal. Chem. 2012, 84, 5556–5564. 10.1021/ac300478f. 22686186 PMC3428216

[ref46] Tu J.; Bennett P. Parallelism experiments to evaluate matrix effects, selectivity and sensitivity in ligand-binding assay method development: Pros and cons. Bioanalysis 2017, 9, 1107–1122. 10.4155/bio-2017-0084. 28737442

[ref47] Wang H.; Chen C. H.; Xiang Z.; Wang M.; Lee C. A convection-driven long-range linear gradient generator with dynamic control. Lab Chip 2015, 15, 1445–1450. 10.1039/C4LC01451K. 25599134

[ref48] Cao L.; Zhang X.; Grimley A.; Lomasney A. R.; Roper M. G. Microfluidic multi-analyte gradient generator. Anal. Bioanal. Chem. 2010, 398, 1985–1991. 10.1007/s00216-010-4168-8. 20835814 PMC2998889

[ref49] Lin F.; Saadi W.; Rhee S. W.; Wang S. J.; Mittal S.; Jeon N. L. Generation of dynamic temporal and spatial concentration gradients using microfluidic devices. Lab Chip 2004, 4, 164–167. 10.1039/b313600k. 15159771

