# Supplementary material for: Everything’s under Control: Maximizing Biosensor Performance through Negative Control Probe Selection
Source: Anal Chem. 2025 Feb 3;97(6):3525–35. doi: 10.1021/acs.analchem.4c05854 (PMC11840803; doi:10.1021/acs.analchem.4c05854)
Supplement: Supplementary file 1 — ac4c05854_si_001.pdf [file ac4c05854_si_001.pdf]

Supporting Information

Everything's under control: Maximizing biosensor performance through negative control probe selection

Joseph Bucukovski[a], Benjamin L. Miller[a,b,c,d,e]

[a] Department of Biochemistry and Biophysics, University of Rochester, Rochester, NY 14627, USA

[b] Institute of Optics, University of Rochester, Rochester, NY 14627, USA

[c] Department of Dermatology, University of Rochester, Rochester, NY 14627, USA

[d] Program in Materials Science, University of Rochester, Rochester, NY 14627 USA

[e] Department of Biomedical Engineering, University of Rochester, Rochester, NY 14627, USA

Item	Description	Page
1.	Supplementary methods	S2
2.	Figure S1: Measurement apparatus	S3
3.	Figure S2: Verification of gradient generator performance	S3
4.	Figure S3: Sensor chip functionalization	S7
5.	Supplementary Table S1: Formulation of reference and capture probes for printing	S8
6.	Figure S4: Assay linearity for trials of CRP detection in 1% BSA background	S9
7.	Figure S5: Assay linearity for trials of CRP detection in serum background with wash buffer or media.	S10
8.	Figure S6: Assay linearity for trials of IL-17A detection in BSA background with wash buffer or media.	S11
9.	Figure S7: Assay linearity for trials of IL-17A detection in serum with EGM-2 media.	S12
10.	Figure S8: R^2 values for all assays	S13
11.	Analysis of candidate control probes by hydropathy index	S13
12.	Figure S9: Peptide and hydropathy analysis	S15

1. Supplementary Methods:

Construction of the gradient generator: This built on an approach previously reported by the Bailey group.[1] Two Fluigent M-switch pumps were used to assemble an apparatus that could deliver a linearly increasing analyte concentration gradient. Both pumps use sine wave mechanical actuation under nitrogen-backed positive pressure to operate. To calibrate the system, separate reservoirs containing either buffer (1x PBS) or concentrated BSA in PBS (8.88 mg/mL) as a mock analyte were fed into pumps 1 and 2, respectively. A program was developed in the Microfluidics Automation Tool software (Fluigent) to set the flow rates for both pumps 1 and 2 and define the linearly increasing or decreasing flow rates over time. For example, pump 1 (buffer) was set to an initial flow rate of 30 µL/min and programmed to decrease by 0.5 µL/min every 5 seconds, while pump 2 (analyte) was set to an initial flow rate of 0 µL/min and programmed to increase by 0.5 µL/min every 5 seconds (Supplementary Figure S2). To reach the set flow rates of the program, the instantaneous flow rate in the pump was reported by the flow rate unit, and the positive pressure in the system was adjusted to enable proper rates in a closed feedback loop. After exiting the flow rate unit, the fluid from both pumps enters the mixing T through either inlet 1 (buffer pump) or inlet 2 (analyte pump). Both fluid fronts undergo complete mixing due to the short diffusion distance between the 2 laminar flow streams. The exiting stream of fluid was sampled every 30 seconds for BSA concentration measurements (A_{280}, Thermo Scientific Nanodrop Lite) for calibration and, for photonic biosensing assays, enters the tubing that interfaces directly with the microfluidic-PIC assembly on the optical stage. The optical system is shown in Figure S1.

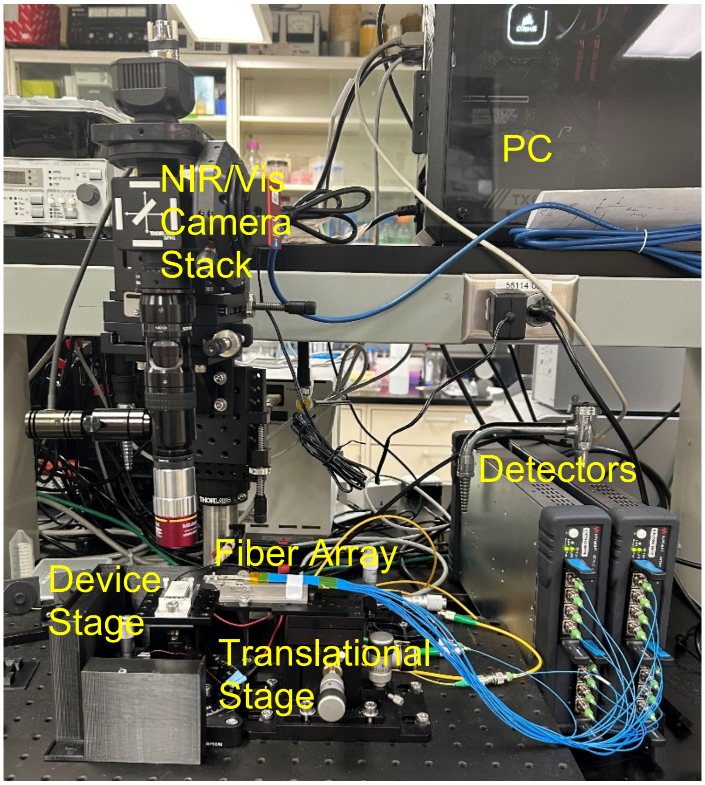

Figure S1: Optical system used for all measurements. A Keysight tunable laser is off camera to the left.

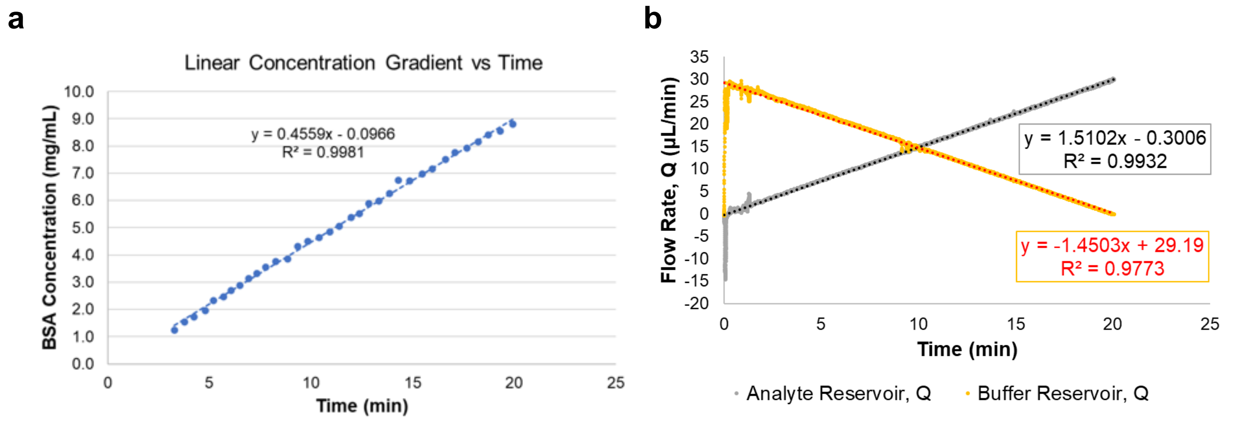

Figure S2: Verification of the linear gradient mixer functionality. (a) Following the 3 trials used to develop the linearity model (Fig 2), this experiment was a verification of the linearity profile produced with the analyte gradient mixer apparatus. Here, the emphasis is on the slope value conforming to those previously observed. Outflowing solution from the mixing T was sampled for A280 measurements to confirm the BSA concentration plotted here as a function of time. The slope (0.4559) was sufficiently close to previous empirical values (Fig 2). (b) The flow rate (Q) metrics recorded by the gradient generator software show the buffer reservoir linearly decreasing while the concentrated analyte reservoir linearly increases over time.

Concentrations were calculated from the gradient system using the following proportions:

(Eq 2a) $\frac{BSA_{stock}}{BSA_{slope}} = \frac{Analyte_{stock}}{Analyte_{slope}}$; (Eq 2b) $\frac{8.877\ mg/mL}{0.452\ mg/mL{\cdot}min} = \frac{0.005\ mg/mL}{CRP_{slope}}$; solve for unknown

(Eq 2c) $CRP_{slope} = 2.55 \times 10^{-4}$ mg/mL·min $\xrightarrow{\text{Convert units}}$ $CRP_{slope} = 4.24 \times 10^{-3}$ μg/mL·sec

(Eq 3a) $\frac{BSA_{stock}}{BSA_{slope}} = \frac{Analyte_{stock}}{Analyte_{slope}}$; (Eq 3b) $\frac{8.877\ mg/mL}{0.452\ mg/mL{\cdot}min} = \frac{0.005\ mg/mL}{IL-17A_{slope}}$; solve for unknown

(Eq 3c) $IL\text{-}17A_{slope} = 5.10 \times 10^{-5}$ mg/mL·min $\xrightarrow{\text{Convert units}}$ $IL\text{-}17A_{slope} = 8.49 \times 10^{-4}$ μg/mL·sec

After determining the rates of change of concentration for each analyte, the expected concentrations (y) could be represented by the following linear equations in the form of y = mx (where x is the time, in seconds):

(Eq 4) $y_{CRP} = 4.24 \times 10^{-3}x$ (Eq 5) $y_{IL\text{-}17A} = 8.49 \times 10^{-4}x$

Photonic sensor fabrication: Sensor photonic integrated circuits (PICs) were fabricated via photolithography using the 300 mm photonic wafer process at AIM Photonics in Albany, NY.[43], [27] Fabricated wafers were supplied on tape after dicing (AIM Photonics TAP Facility, Rochester, NY). Individual 5.8 x 4.0 mm PICs used in this work contain two ring banks, 14 ring resonators per bank, with two rings per waveguide (Figure 1c and Supplementary Figure S3 a, b). Near-IR light was coupled into the waveguides of the PIC using a 16-channel fiber array. An on-chip seven-way splitter allowed light to be coupled into the input waveguide from each ring bank, while simultaneously measuring the power from 7 output channels.

Preparation of PhRR sensors: PICs were treated with piranha solution (1 part 30% hydrogen peroxide to 3 parts sulfuric acid; *Caution:* piranha solution is highly caustic and reacts violently with organic materials) for 30 minutes under agitation to remove organic debris and residual photoresist from the sensor and to activate surface hydroxyl groups of native SiO_2 for subsequent silanization. After several washes in 18-MΩ (Nanopure) water, the PICs were dried under a stream of nitrogen. Next, all substrates were transferred into a chemical vapor deposition tool (Yield Engineering Systems 1224P) in the cleanroom for functionalization with 3-Glycidyloxypropyl)trimethoxysilane (GPTMS) to produce an amine-reactive linker film (Supplementary Figure S3, e). Spectroscopic ellipsometry (Woolam alpha-SE) was used to measure film layer thicknesses on sentinel Si/SiO_2 chips. The mean film thickness on sentinel Si/SiO_2 chips was determined to be 4.5 Å. If further functionalization was not conducted immediately, PICs were stored in a Mylar bag with desiccant following several fill-purge cycles with dry nitrogen.

Capture and reference probe formulation: Prior to PhRR sensor spotting, anti-FITC and anti-IL-17A were buffer-exchanged and concentrated into 1x PBS at pH 7.4 using Amicon centrifugation filters (EMD Millipore). This step was needed to remove the sodium azide preservative from the anti-FITC and anti-IL-17A stock, as it interferes with amine-reactive immobilization chemistry. Other probes did not have this additive and therefore did not need this step. Multiple formulations were tested for each capture and reference probe mainly by altering the pH and including additives (such as trehalose) to optimize ring trench spotting coverage. The immobilization density was controlled by keeping the concentrations consistent across each probe on the ring resonator sensors. Optimized formulations printed for each sensor probe, and used for all sensing experiments described here, are provided in Supplementary Table S1.

To functionalize PICs with proteins, amine-reactive sensor rings were spotted with protein solution based on the formulations given in Table S1 using a Scienion SX piezoelectric

microarrayer (Scienion, A.G.). Each ring received 8 droplets of probe solution at approximately 330 pL each as measured by the instrument, resulting in a total volume of 2.6 nL per ring. Head camera images taken with the piezoelectric microarray tool revealed nearly complete coverage of each ring resonator with probe with little to no merging of solutions (Supplementary Figure S3, c). The final printing layout is shown in Supplementary Figure S3, d; ring banks were duplicated on every PhRR PIC. Capture antibody and negative control probe solutions were allowed to immobilize onto the sensors for 1.5 hours at 80% relative humidity. After covalent tethering of the proteins, the sensors were rendered shelf-stable by printing 9 spots of Stabilcoat Plus (Surmodics) onto the rings. The stabilizer was allowed to dry onto the protein-coated rings for 1 hour at 50% relative humidity.

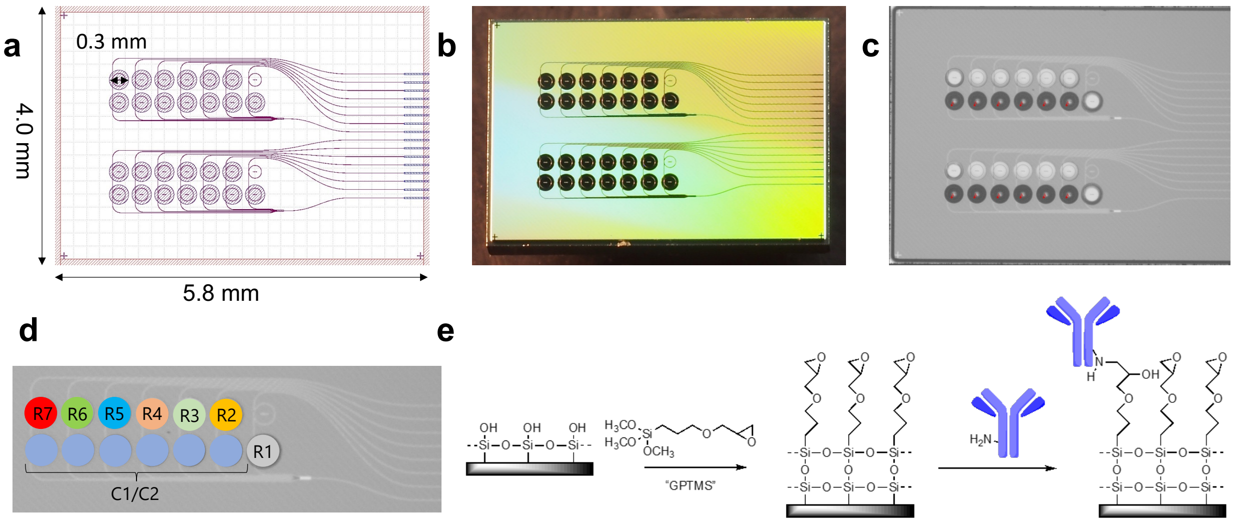

Figure S3: Functionalization of photonic ring resonator biosensor PICs. (a) Design file of the PIC used for wafer-scale fabrication. The overall dimensions are 4.0 x 5.8 mm with each ring resonator trench measuring 300 µm in diameter. (b) Photograph of an actual PhRR PIC. (c) Image captured by the SX piezoelectric microarrayer head camera demonstrating complete filling of the ring resonator trenches with spotting solution and no merging of individual probes. The darker trenches indicate liquid that was more recently deposited. (d) Printing layout of the sensors using the probe ID from Table S1. Capture probes are colored in light blue, while negative controls are colored differently. (e) Functionalization scheme of the PhRR sensors. GPTMS linker chemistry was deposited in a thin film using CVD instrumentation, followed by amine-reactive immobilization of protein probes after spotting.

Ring ID	Protein	Probe Concentration (mg/mL)	pH	Additives
R1	BSA	0.933	7.4	0.00027% Tween-20
R2	Mouse IgG1 isotype control	0.933	7.4	1.0% Trehalose
R3	Mouse IgG2a isotype control	0.933	7.4	1.0% Trehalose
R4	Mouse IgG2b isotype control	0.933	7.4	1.0% Trehalose
R5	Anti-FITC, goat polyclonal	0.933	7.4	None
R6	Rat IgG1 isotype control	0.933	7.4	1.0% Trehalose
R7	Cytochrome c	0.933	7.4	None
C1	Anti-IL-17A, mouse monoclonal	0.933	7.4	None
C2	Anti-CRP, mouse monoclonal	0.933	7.2	2.5% Trehalose

Supplementary Table S1: Formulation of reference and capture probes used for sensor printing. Formulations were developed in two ways: (1) based on previous work with these analytes (typically optimized using Arrayed Imaging Reflectometry, a label-free reflectometric sensor developed in our group) following a procedure analogous to that described in: Mace et al., 2008;[2] (2) based on additives present in the lyophilized antibodies or proteins as obtained from the supplier. For example, if the protein was supplied as a lyophilizate with trehalose (such as was the case for anti-IL-17A), it was simply reconstituted in PBS.

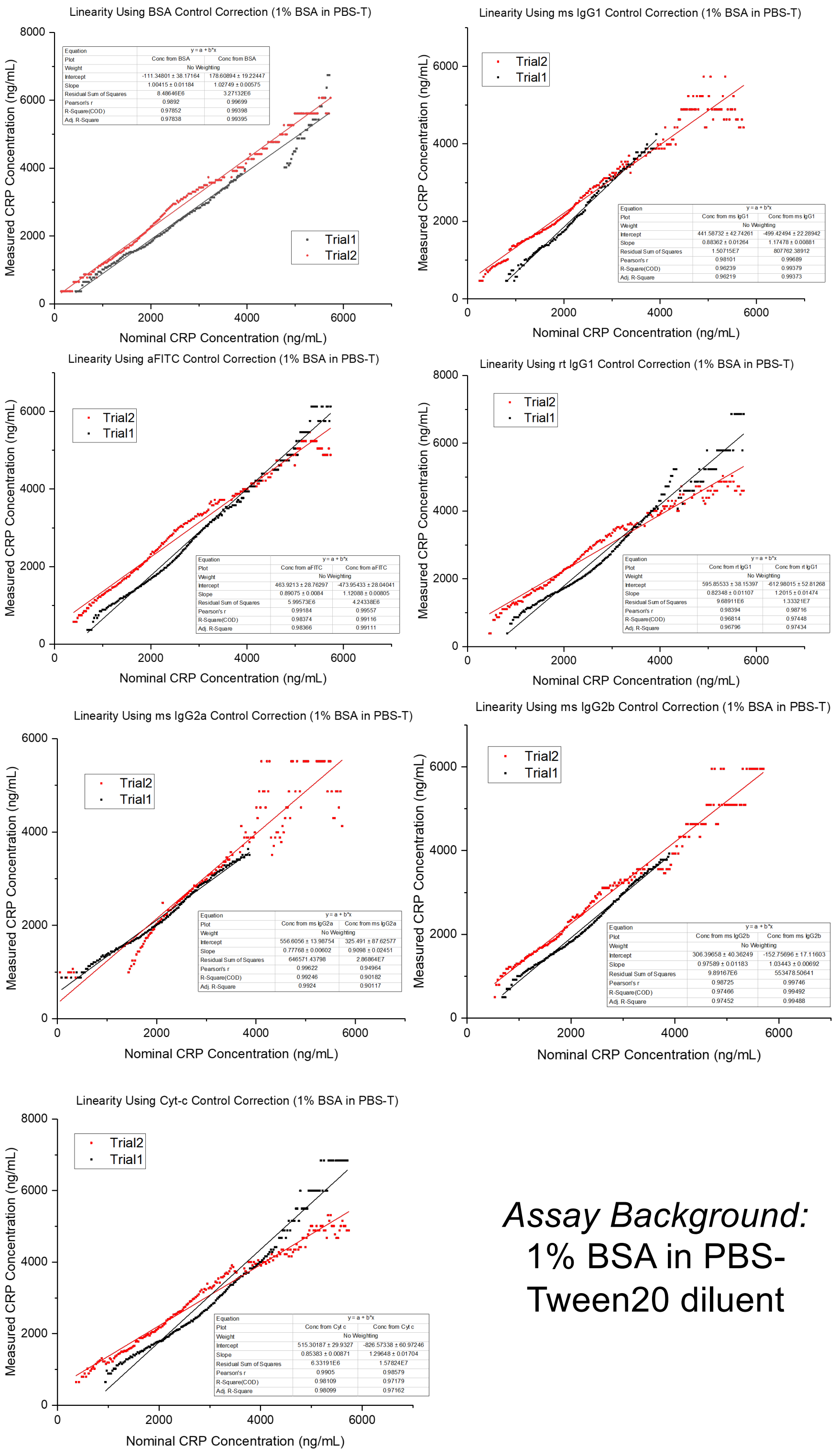

Figure S4: Assay linearity for trials of CRP detection in 1% BSA background. (a) Measured CRP concentration determined by calibration curve shift interpolation was plotted against the nominal (expected) CRP concentration. Linear fits were produced to determine the slope (m), y-intercept (b), and goodness of fit value (R-squared).

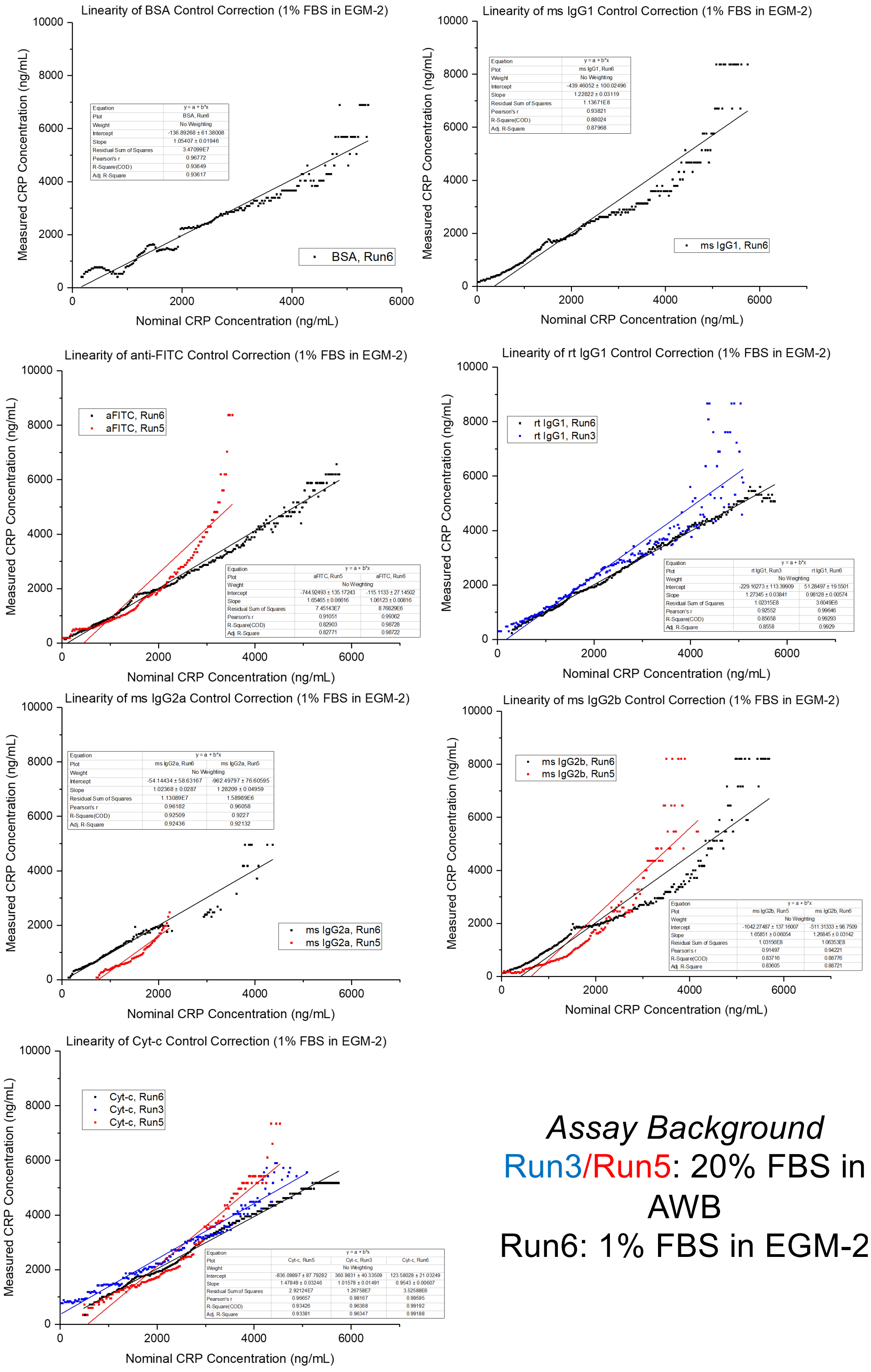

Figure S5: Assay linearity for trials of CRP detection in serum background with wash buffer or media. (a) Measured CRP concentration determined by calibration curve shift interpolation was plotted against the nominal (expected) CRP concentration. Linear fits were produced to determine the slope (m), y-intercept (b), and goodness of fit value (R-squared).

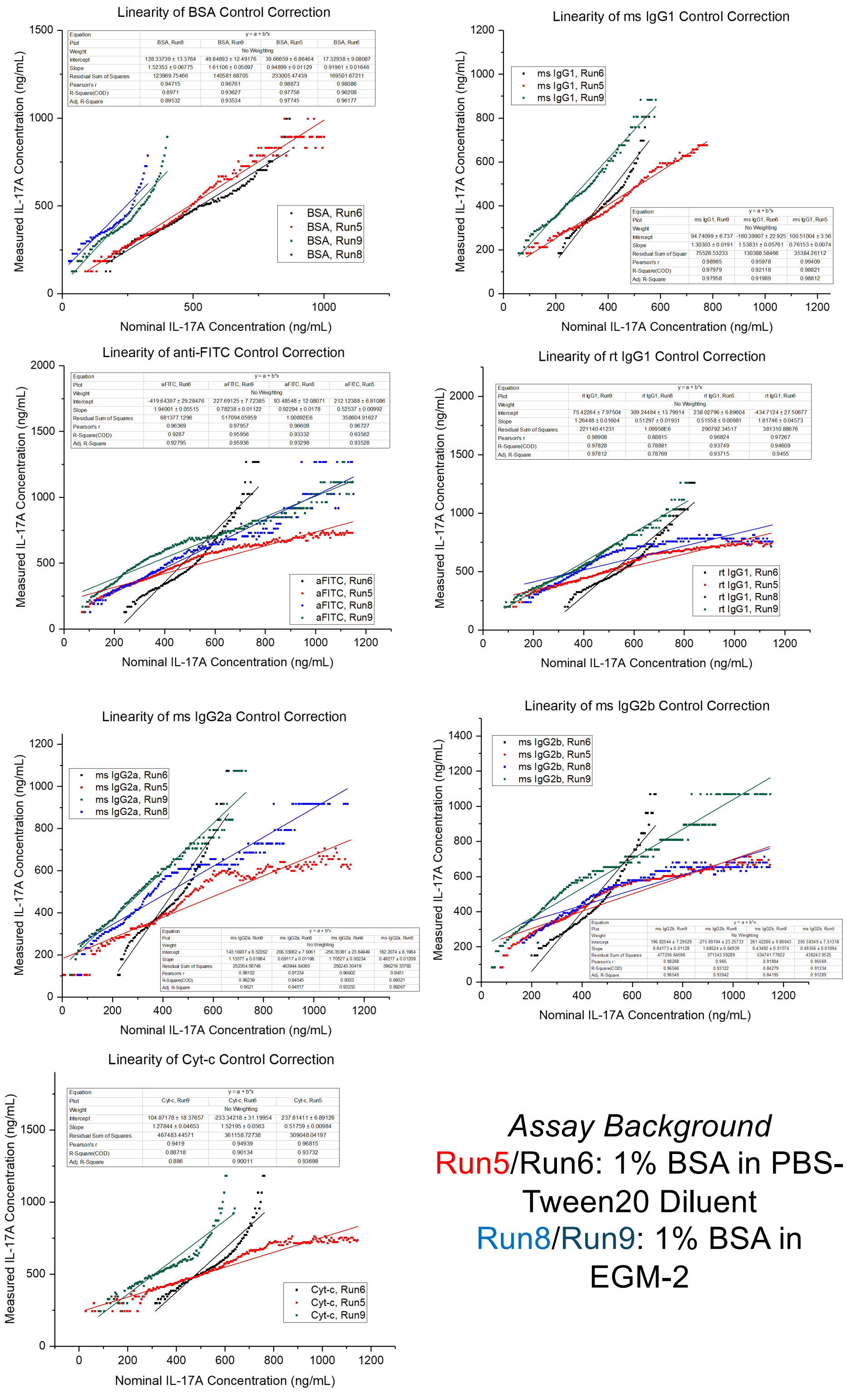

Figure S6: Assay linearity for trials of IL-17A detection in BSA background with wash buffer or media. (a) Measured IL-17A concentration determined by calibration curve shift interpolation was plotted against the nominal (expected) IL-17A concentration. Linear fits were produced to determine the slope (m), y-intercept (b), and goodness of fit value (R-squared).

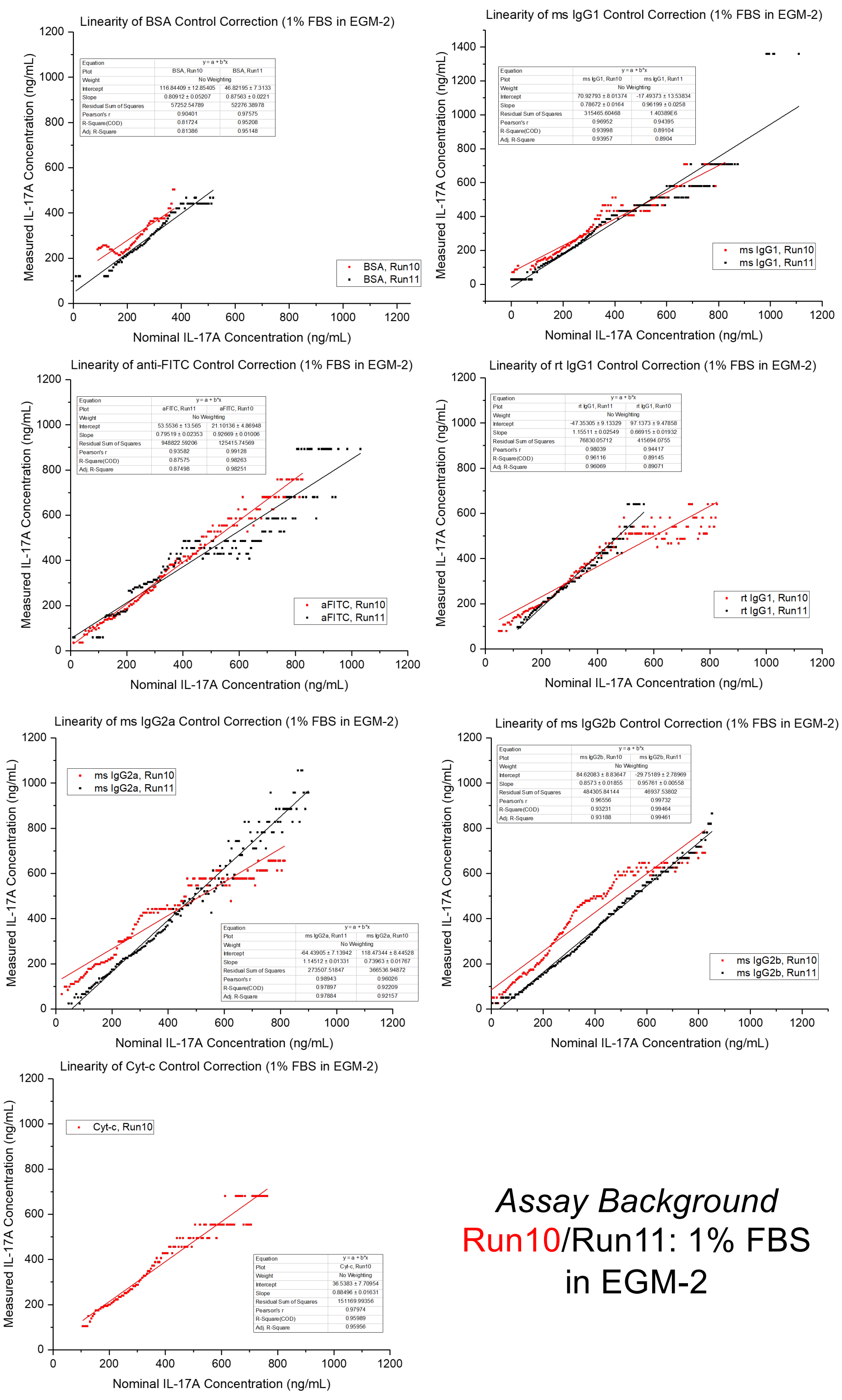

Figure S7: Assay linearity for trials of IL-17A detection in serum with EGM-2 media. (a) Measured IL-17A concentration determined by calibration curve shift interpolation was plotted against the nominal (expected) IL-17A concentration. Linear fits were produced to determine the slope (m), y-intercept (b), and goodness of fit value (R-squared).

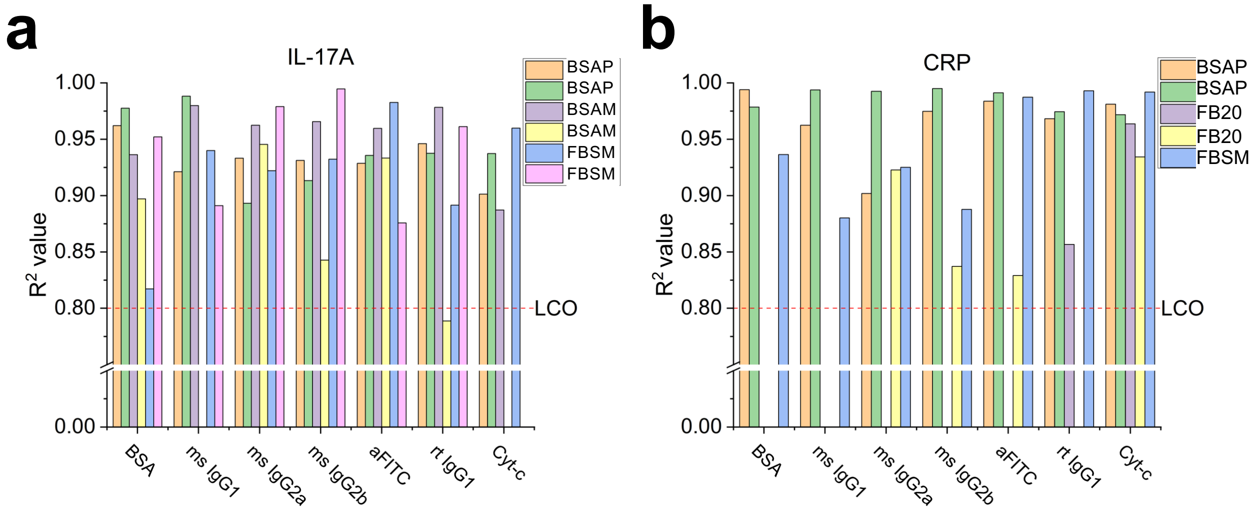

Figure S8: R^2 values for all assays.

Analysis of candidate control probes by hydropathy index:

Investigating the role of nonspecific binding (NSB) in optimizing PhRR assay performance was a major driver of this work. We hypothesized that matching the isotype between the capture antibody and negative control probe could faithfully subtract nonspecific interactions from the overall detection (Fig 1b). To study this, non-conserved regions of mouse and rat antibody isotypes were identified in the hinge region. The amino acid sequences of mouse and rat IgG isotypes were previously aligned by R. Nezlin (Fig S9, a).[3] We posit that this sequence divergence may be responsible for the differences in nonspecific binding observed across various isotypes since the rest of the IgG molecule is highly conserved. To further investigate, the hydropathy index was computed and plotted as a function of isotype and hinge position (upper, core, and lower hinge). The hydropathy index is a number value assigned to an amino acid side chain which describes its hydrophilic or hydrophobic nature where the more positive the value, the more hydrophobic the interaction with surrounding water molecules. In general, the upper and lower hinge regions were hydrophilic in character across both species and isotypes, while the core region contained amino acid side chains which were slightly hydrophobic (isoleucine) for both

mouse and rat IgG1 (Fig S9, b). This apparent hydrophobicity is likely to resist nonspecific binding due to electrostatic interactions, particularly those of serum albumin proteins which are negatively charged at physiological (neutral) pH. It is important to consider not only the protein characteristics of each probe at an amino acid level, but also the clonality of the antibody probe in question and the molecular weight of non-antibody reference controls. Both capture antibodies used in this study were monoclonal antibodies, as were the four isotype controls (Fig S3, d and Table S1). Goat anti-fluorescein (FITC) is a polyclonal antibody, meaning that it recognizes more than one epitope on the FITC immunogen. Although FITC is not found endogenously in any biological system, it is reasonable to expect that its polyspecificity would lead to higher levels of nonspecific binding.[13] With regard to non-antibody probes, such as BSA and cytochrome c, the molecular weight of the protein will dictate the degree of steric crowding that is observed on the surface of a functionalized ring resonator sensor. Steric crowding, in this context, prevents matrix constituents (serum globulins, hormones) from wedging in between two adjacent probe molecules and remaining on the sensor either electrostatically, or perhaps covalently bound to the underlying silane linker. Therefore, due to the size of BSA (66.4 kDa) compared to the size of cytochrome c (~12 kDa), we would anticipate that BSA is a superior choice to resist the aforementioned mode of nonspecific binding by providing increased steric repulsion to off-target proteins.

a

Protein	Upper Hinge (aa216)	Core	Lower Hinge (aa238)
Mouse IgG1	VPRDCG	CKPCIC	TVPSEVS
Mouse IgG2a	EPRGPTIKP	CPPCKC	PAPNLLGGP
Mouse IgG2b	EPSGPISTINP	CPPCKECHKC	PAPNLEGGP
Rat IgG1	VPRNCGGD	CKPCIC	TGSEVSS

b

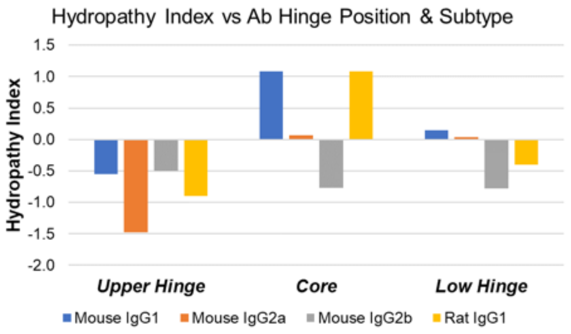

Figure S9: (a) Table showing the amino acid compositions in the upper, core, and lower hinge regions between different isotypes of mouse and rat IgG antibody. (b) Bar graph showing the variations in hydropathy index between the specific hinge regions of rat and mouse IgG isotypes.

References:

[1] M. T. Marty, C. D. K. Sloan, R. C. Bailey, and S. G. Sligar, "Nonlinear analyte concentration gradients for one-step kinetic analysis employing optical microring resonators," *Anal Chem*, vol. 84, no. 13, pp. 5556–5564, Jul. 2012, doi: 10.1021/ac300478f.

[2] Mace, C. R.; Yadav, A. S.; Miller, B. L. "Investigation of non-nucleophilic additives for reduction of morphological anomalies in protein arrays" *Langmuir*, **2008**, *24*, 12754-12757

[3] R. Nezlin (Ed.), Chapter 1 – General Characteristics of Immunoglobulin Molecules, Academic Press, New York (1998), pp. 3-73
